# Cell wall mechanical stress could coordinate septal synthesis and scission in *Staphylococcus aureus*

**DOI:** 10.1128/mbio.01728-25

**Published:** 2025-10-13

**Authors:** Sheila Hoshyaripour, Marco Mauri, Jamie K. Hobbs, Simon J. Foster, Rosalind J. Allen

**Affiliations:** 1 Theoretical Microbial Ecology, Institute of Microbiology, Faculty of Biological Sciences, Friedrich Schiller University Jena 9378, Jena, Germany; 2 Cluster of Excellence Balance of the Microverse, Friedrich Schiller University Jena 9378, Jena, Germany; 3 Department of Physics and Astronomy, University of Sheffield 152815, Sheffield, United Kingdom; 4 The Florey Institute for Host-Pathogen Interactions, University of Sheffield, Sheffield, United Kingdom; 5 School of Biosciences, University of Sheffield 152813, Sheffield, United Kingdom; Case Western Reserve University School of Medicine, Cleveland, Ohio, USA

**Keywords:** *Staphylococcus aureus*, cell wall mechanical stress, cell division, cell cycle, peptidoglycan hydrolases

## Abstract

*Staphylococcus aureus* divides by building a septum and then splitting into two daughter cells. Scission should be coordinated with septum completion to avoid cell lysis; however, it is not known how this is achieved, or what the relative roles of mechanical forces and the activity of peptidoglycan hydrolase enzymes are. Here, we show using thin-shell mechanics that septum formation causes a localized decrease in mechanical stress at the cell’s equator. We propose that this local decrease in stress could act as a mechanical trigger for hydrolase activity, leading eventually to splitting. This mechanical trigger model can explain observed cell division defects, including premature splitting and failure to initiate splitting. The model also shows how cell size, turgor pressure, cell wall thickness and stiffness, and the relative rates of synthesis and hydrolysis combine to determine cell cycle timing and the outcome of antibiotic exposure. Bacterial cell division requires dynamic orchestration of molecular players, in concert with cell wall mechanics. Our work suggests how mechanical forces could coordinate with enzyme activity in the control of this complex process.

**IMPORTANCE:**

*Staphylococcus aureus* is a major threat due to its ability to generate antibiotic-resistant strains. Understanding *S. aureus* division is therefore of great importance, but we do not know how septum formation is coordinated with cell scission. Previous works have shown that both mechanical stress and autolysin activity play key roles in scission, but it is unclear how mechanical and biochemical cues work together. Here, we propose a “mechanical trigger” model for the interplay between mechanical stress and autolysin activation. We use mathematical modeling to show that stress decreases in the *S. aureus* cell wall close to the division site as the septum is formed, and we propose that this could trigger autolysin activity. Our model explains reports of diverse division outcomes in the presence of mutations and antibiotics and points to a general link between cell geometry and antibiotic resistance.

## INTRODUCTION

Cell division is a fundamental biological process that requires dynamic control of the physical and biochemical activities of multiple molecular players. Cell division has been intensively studied in gram-negative bacteria (1, 2), but less is known for gram-positive organisms, especially concerning the final stage of division. *Staphylococcus aureus* is a gram-positive facultative pathogen; its ability to generate antibiotic-resistant variants, such as methicillin-resistant *S. aureus* (MRSA), makes it clinically important (3). *S. aureus* cells are spherical, with high turgor pressure and a thick (∼ 20 nm) peptidoglycan (PG) cell wall (4). To divide, *S. aureus* synthesizes an internal septum that divides the cell into two pseudo-hemispheres before splitting; the latter event takes less than 2 ms (5, 6). The timing of splitting should be coordinated with septum formation, since premature splitting, before the septum is complete, leads to cell death (7–9). However, it is not known how this coordination is achieved. One possibility is a “septum completion signal” mechanism, in which completion of the septum triggers hydrolase activation via a signal. It has been suggested that PBP1, a key enzyme in septal synthesis (10), might function as this signal (11, 12), but recent work shows that division can happen without the transpeptidase activity of PBP1 (13). It has also been observed that stalling of septum formation can lead to premature splitting (8, 9), suggesting that the cell may actually commit to splitting before the completion of septum formation.

**Fig 1:**
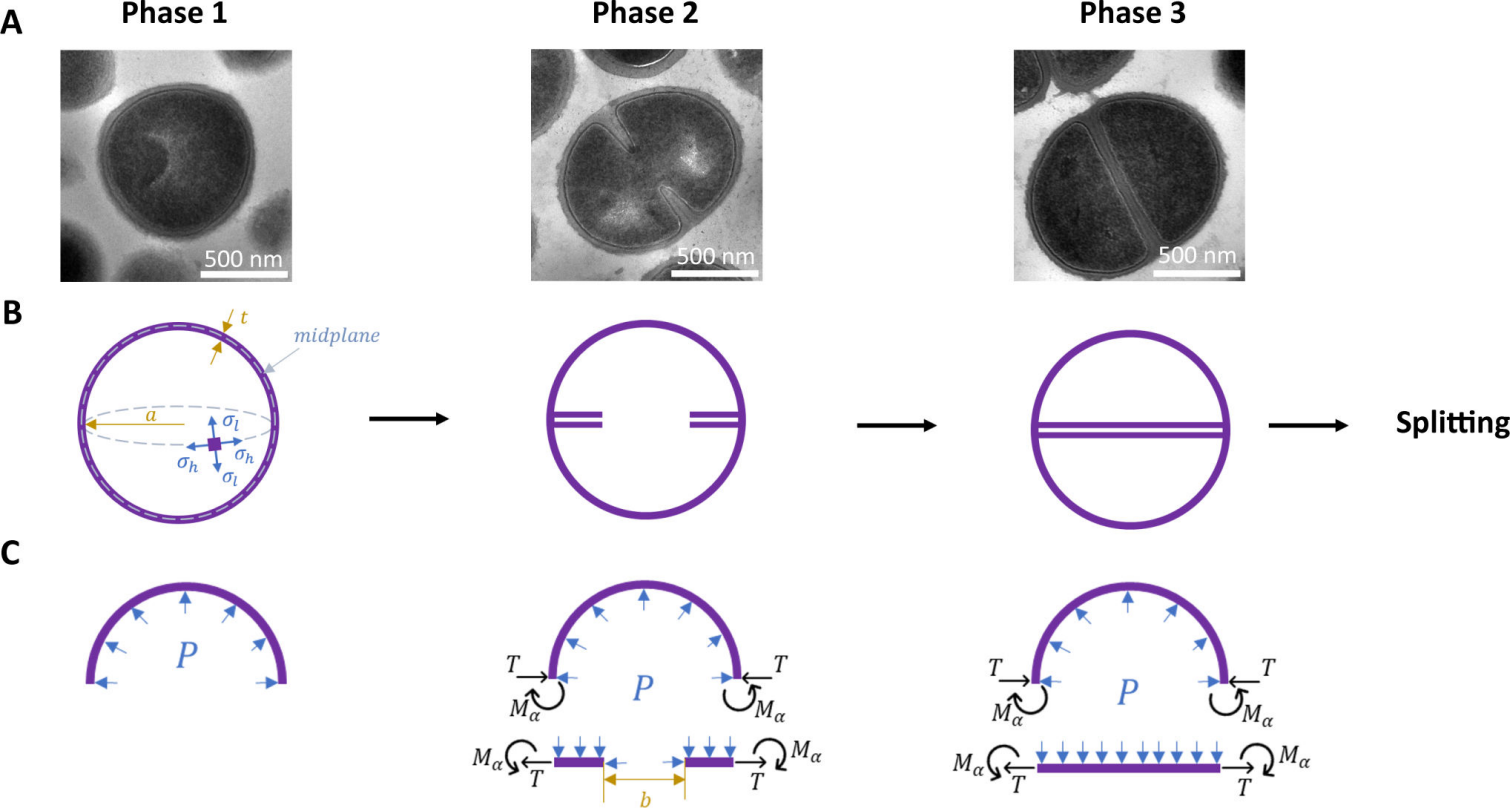
Modeling *S. aureus* cell cycle. (**A**) Transmission electron microscopy (TEM) images illustrating the stages of the *S. aureus* cell cycle, (**B**) Illustration of *S. aureus* cell cycle phases in the model. Phase 2 starts with the onset of septum synthesis and ends when the septum is complete. Phase 3 describes the period between septum completion and cell splitting. (**C**) Illustration of the principles underlying the calculation of mechanical stress, via force diagrams for the different cell cycle phases. In phase 1, turgor pressure exerts a uniform outward force on the cell wall. In phases 2 and 3, the presence of the septum and the continuity of the material lead to an additional force (*T*) and bending moment (*M_α_*) that change the local stress pattern. *T* and *M_α_* are dependent, and *T* depends on the aperture size (*b*), pressure (*P*), and the mechanical and geometrical properties of the cell wall and the septum (see Methods section for details). The TEM images in A were kindly provided by Dr. Lucia Lafage, University of Sheffield.

Hydrolase enzymes, which cleave bonds in the PG cell wall, play a key role in *S. aureus* growth and division (7). Autolysins are hydrolases that aid cell splitting by gradually digesting the peripheral ring of PG that holds together the two nascent daughter cells (14) (Fig. 1); two of the major autolysins in *S. aureus* are Atl and Sle1. Loss of these enzymes leads to cell division defects (15, 16), but we lack a full picture of how their activity is controlled during the cell cycle (17). Hydrolases are known to be regulated via transcriptional control, protein localization, proteolysis, chemical modification of the PG, and interaction with other proteins, salt, or lipoteichoic acids (18, 19).

Mechanical forces are also believed to play a central role in cell division. The high turgor pressure in *S. aureus* is expected to generate strong mechanical stresses in the PG cell wall, which change during the cell cycle. Pioneering previous work showed that the rapidity of the splitting event could be explained by mechanical failure of the part of the cell wall that forms a ring peripheral to the septum and subsequent crack propagation (5). Mechanical failure of the peripheral PG was ascribed to a possible increase of stress in the peripheral ring prior to splitting due to a lack of growth in this part of the PG relative to the neighboring parts of the cell wall (5). However, the picture is as yet incomplete since the role of hydrolases remains unclear (17), and it is hard to measure local mechanical stresses and the growth and breakage dynamics of PG in the peripheral ring with current techniques.

The roles of mechanical forces and PG hydrolysis may be linked through mechanical stress regulation of hydrolase activity. This concept has a long history, having been postulated by Koch almost 30 years ago as a mechanism for achieving cell wall homeostasis via the coordination of PG hydrolysis throughout the thick cell wall with PG synthesis at the inner wall surface (20). Koch’s model suggests that growth hydrolases, which allow the PG to expand laterally during growth, should be upregulated by stress (20), but early indications in *Bacillus subtilis* suggest that turnover hydrolases, which cleave PG on the outer edge of the cell wall, are activated under low stress conditions (21). The *S. aureus* autolysin Atl, which is central in cell splitting, may also be activated by low stress. In live cells (where PG is stressed by turgor), Atl is present throughout the cell cycle. It is localized to the division site by wall teichoic acids (that prevent its binding to other parts of the cell wall) but is only activated at the peripheral ring during cell division (15, 22, 23); however, Atl-mediated cell lysis can be triggered by loss of turgor due to exposure to detergent, and Atl can also rapidly hydrolyze isolated, and therefore unstressed, PG (24).

In this work, we propose an extended biophysical model for the role of mechanical stress in the initiation of cell scission. Our "mechanical trigger" model is based on mechanical-stress regulation of PG hydrolase activity. Using a thin-shell mechanical model, we predict how the pattern of mechanical stress in the cell wall changes through the *S. aureus* cell cycle, finding that septum formation causes a decrease in local circumferential stress at the cell equator close to the division site. We propose that this local stress decrease could act as a trigger for autolysin activity, leading to mechanical failure in the peripheral PG ring and ultimately scission. We show that this mechanical trigger model can explain diverse observations of division defects, including premature splitting and failure to split, and predicts how division timing and success depend on cell size, turgor, the mechanical properties of the cell wall, and enzyme activities. Our model could help explain how hydrolase activity and mechanical forces work together to orchestrate successful cell division, with implications for understanding antibiotic susceptibility and resistance.

## RESULTS

### Septum formation decreases mechanical stress close to the division site

To predict how the pattern of mechanical stress in the cell wall changes during the *S. aureus* cell cycle, we used a solid elastic model (Fig. 1). In phase 1 of the cell cycle, before septum formation starts, the cell wall is modeled as a spherical shell of elastic material that is inflated by turgor pressure. In phase 2, during septum formation, we model the cell as two pseudo-hemispheres, each of which is connected to a disc with an aperture, representing the incomplete septum. In phase 3, the septal disc is complete.

For each cell cycle phase, we can calculate the distribution of mechanical stress in the midplane of the cell wall in the longitudinal (meridional) and circumferential (hoop) directions (these stresses are denoted $\sigma_{l}$ and $\sigma_{h}$, respectively). In phase 1, the stress in the cell wall is uniform and equal in the two directions, and its magnitude scales with the turgor pressure *P* and the ratio of the cell radius *a* in the depressurized state to the cell wall thickness *t*:


(1) $$\sigma_{l}\left(phase\;1\right)=\sigma_{h}\left(phase\;2\right)=\sigma =\frac{Pa}{2t}$$

In phases 2 and 3, the longitudinal stress $\sigma_{l}$ remains unchanged in the midplane, but the septum causes the circumferential stress to decrease at the cell equator, where the cell wall meets the septum:


(2) $$\sigma_{h}\left(phases\;2\;and\;3,\;equator\right)=\frac{Pa}{2t}-\frac{T\lambda }{t}$$

where $\lambda =\sqrt[{4}]{3\left(1-\nu^{2}\right){\left(\frac{a}{t}\right)}^{2}}$, *ν* is Poisson’s ratio and *T* depends not only on turgor pressure, cell size, and wall thickness but also on the septum aperture size (*b*) and on the relative stiffness of the septum and the cell wall. The circumferential stress at the equator is minimal after septum completion, that is, in phase 3. Full expressions for the distribution of stress in the cell wall in different cell cycle phases are given in the Methods section.

To predict cell wall stress quantitatively, we use parameter values corresponding to the methicillin-sensitive (MSSA) *S. aureus* strain SH1000: cell volume *V_cell_ =* 1.22 µm^3^ (which we here assume remains constant during the cell cycle, although see Supplementary Material, Section V and VI), cell wall thickness *t* = 20 nm (13) (assumed to be the same for septum and outer wall), and turgor pressure *P* = 2 MPa (4) (assumed to remain constant through the cell cycle[25]). We assume that the ratio *E_r_* of the Young’s modulus of the septum to that of the peripheral cell wall is 1.2, consistent with measurements of the stiffness of newly exposed septal material versus older cell wall (25), and with observations that the newly synthesized septum consists of a dense PG mesh with tightly packed concentric rings, while the outer wall has a dense mesh transitioning to an open mesh structure (26). Although electron microscopy evidence suggests that the growing septum is wedge-shaped (27), we here assume for simplicity that the septum is locally uniform in thickness but the thickness increases gradually as the aperture decreases throughout phase 2 of the cell cycle (for calculations with a wedge-shaped septum see Supplementary Information, Section IV). We use a Poisson’s ratio ν = 0.499, as is common for biological materials (28).

**Fig 2:**
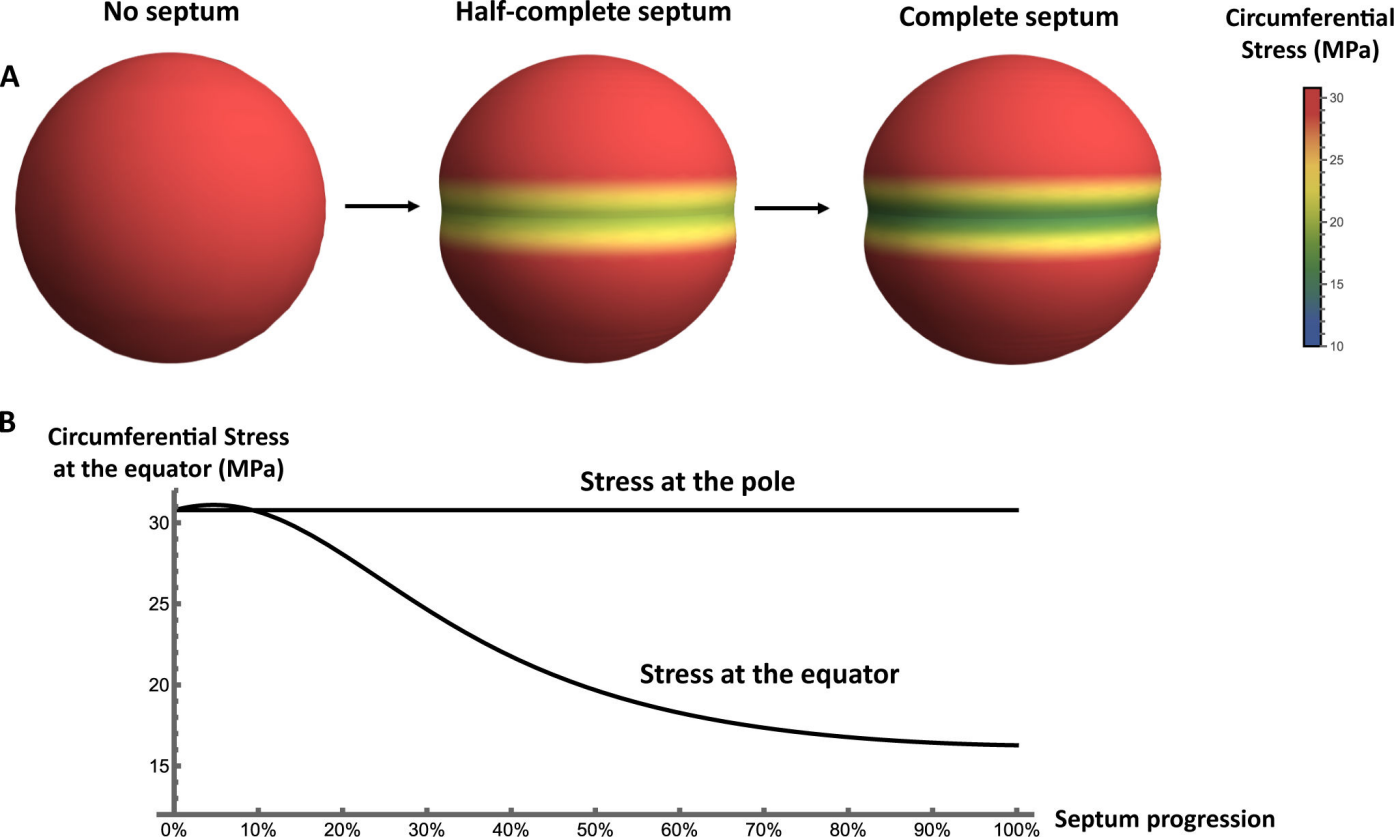
Mechanical stress in the *S. aureus* cell wall during the cell cycle. (**A**) As the cell cycle progresses, stress at the equator decreases due to growth of the septum while the stress remains unchanged at the pole. The stress distributions shown here were calculated for MSSA with cell volume 1.22 µm^3^, thickness 20 nm, turgor pressure 2 MPa, and septum relative stiffness 1.2. (**B**) Graphical representation of the changes in circumferential stress at the cell pole and equator as septum synthesis progresses. Here, septum progression is defined as (*a − b)/a,* where *a* is the cell radius and *b* is the septum aperture size, which decreases as the septum is formed (note that *a* and *b* are defined for the unpressurized cell; see Supplementary Information, Section II).

Calculating the circumferential stress in phases 1, 2 and 3 of the cell cycle reveals a striking pattern: the circumferential stress within the cell wall is around 30 MPa at the cell poles but decreases strongly at the cell equator as the septum progresses during phase 2, eventually reaching ∼16 MPa in phase 3, about half its value in phase 1 (Fig. 2). Intuitively, the presence of the septum restrains the deformation of the cell wall at the point where septum and cell wall meet. Therefore, the outward deformation of the cell wall is smaller in the presence of the septum than it would be in the absence of the septum, resulting in lower mechanical stress in the cell wall material. Interestingly, our model also predicts a characteristic stress pattern in the septum itself: here, the tangential stress shows a maximum at the inner edge of the growing septum, whereas the radial stress shows a different pattern, with the septum material being radially compressed at its inner edge but under tension at its outer edge (see Supplementary Information, Section III).

We also extended our calculations to account for the increase in cell size due to growth and the fact that *S. aureus* cells are actually somewhat ellipsoidal and become more elongated through the cell cycle (6), using measured values for *S. aureus* cell dimensions in phase 1 and phase 3 (6). For a growing sphere, we still predict the reduction of stress around the division site in phase 3 (Fig. S7). For an ellipsoidal cell in phase 1 (without a septum), the circumferential stress is lower at the cell poles than at the equator. However, our calculations for an ellipsoidal, elongating cell produce similar results, with an emergent band of lower circumferential stress around the division site as the septum is formed (see Supplementary Information, Sections V and VI).

### Mechanical trigger model for the coordination of septum formation with cell splitting

We hypothesized that the striking decrease in circumferential stress at the cell equator as the septum is synthesized (Fig. 2) could act as a signal for hydrolase activation around the division site. In this "mechanical trigger" model, autolysins involved in cell splitting (including Atl and/or Sle1) activate during phase 2 when the circumferential stress around the division site has decreased to a threshold value, which we call $\sigma^{*}$. The time in phase 2 at which this triggering event happens is determined by the changes in mechanical stress at the cell equator (Fig. 2) and therefore corresponds to a different amount of septum progression, depending on the physical parameters of the cell. Splitting then occurs a fixed time later, which is the time needed for the autolysins to digest the peripheral ring of PG.

**Fig 3:**
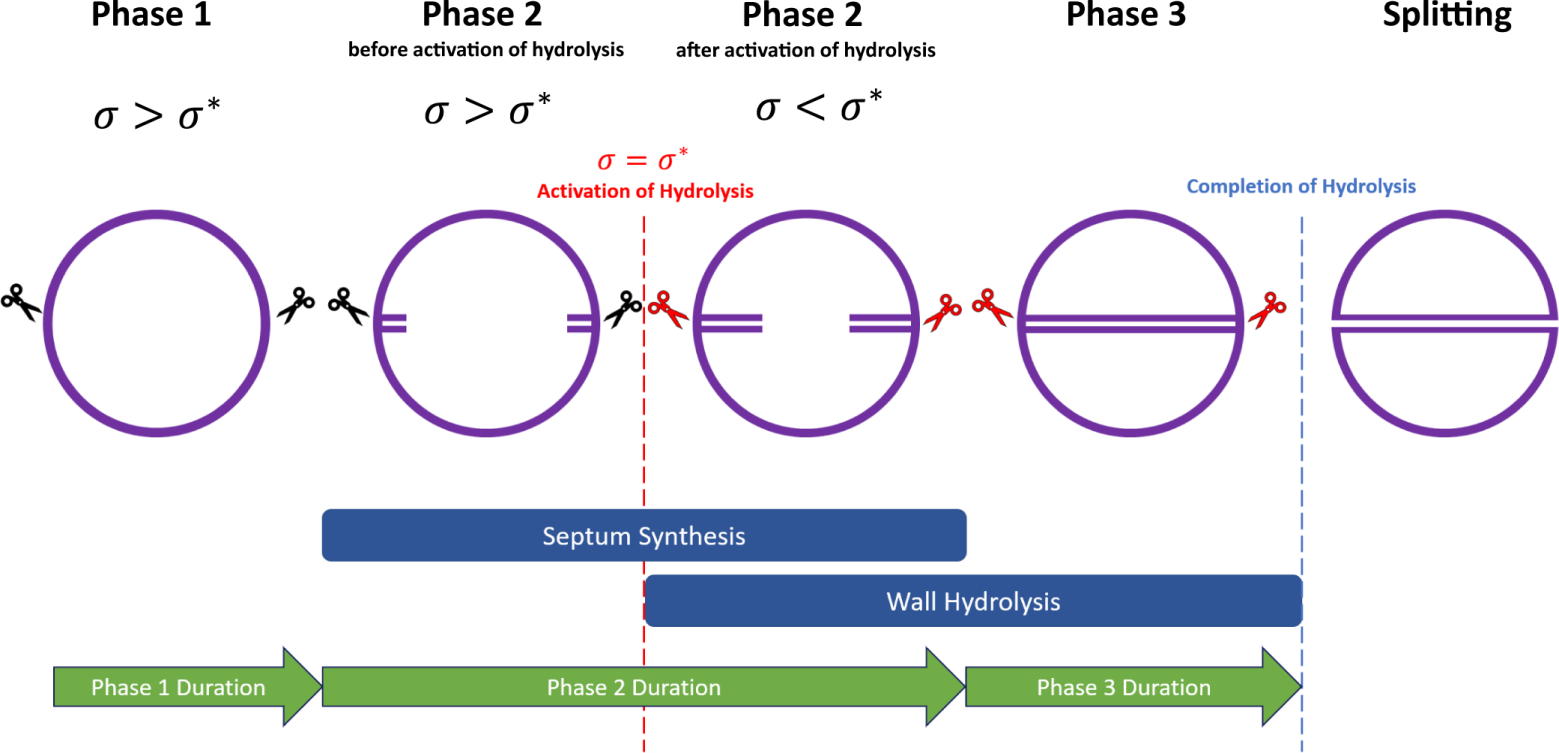
Mechanical trigger model for coordination of *S. aureus* septum synthesis and cell splitting. In a cell cycle leading to successful division, circumferential stress at the equator in phase 1 is higher than the threshold stress $\sigma^{*}$. Autolysins (indicated by scissors) are present at the division site but are inactive (black scissors). During phase 2, the septum is synthesized, and the circumferential stress gradually decreases at the equator. At a particular time point in phase 2, the stress at the equator becomes equal to $\sigma^{*}$ and the autolysins are activated (red scissors). From now on, the synthesis of the remaining part of the septum proceeds in parallel with cleavage of the peripheral ring. Once septum synthesis is complete, phase 3 starts. Phase 3 ends when peripheral ring cleavage is complete and the cell splits into two daughter cells.

In the mechanical trigger model, a successful cell cycle, with division, proceeds as follows (Fig. 3). In phase 1, the stress at the equator is higher than the threshold stress $\sigma^{*}$. Autolysins are present at the division site (possibly localized by wall teichoic acids (15)), but they are not active. At the start of phase 2, the septum starts to be synthesized, which causes the stress to decrease at the cell equator. At a time point in phase 2 that depends on the physical properties of the cell and on the environmental conditions, the stress reduces to the threshold value $\sigma^{*}$and the autolysins involved in cell splitting are activated. Thereafter, completion of septum synthesis and cleavage of the peripheral ring happen in parallel. After the septum is complete, autolysin activity continues until the peripheral ring is cleaved, the cell splits, and the two hemispherical daughters remodel their shape and enter a new phase 1. In this model, the duration of phase 3 is determined by the time needed for completion of peripheral ring cleavage once the septum is complete (Fig. 3).

### High-level resistant MRSA strains are predicted to have lower cell wall stress and trigger autolysin activity earlier in the cell cycle

To understand the implications of the mechanical trigger model for different *S. aureus* strains, we repeated our calculations for parameters corresponding to a well-characterized MRSA strain, *mecA*^+^ rpoB^*^, which shows high-level resistance to the antibiotic methicillin (13, 29). This MRSA strain carries the *mecA* gene, encoding the PBP2a transpeptidase, and an rpoB^*^ mutation in RNA polymerase (13, 29). Methicillin inhibits the activity of the two essential PG synthesis enzymes, PBP2 and PBP1, leading to cell death via lysis (7, 10) (while also binding PBP3 and PBP4 with low affinity (30)). In the *mecA*^+^ rpoB^*^ strain, PBP2 transpeptidase activity is replaced by PBP2a, whereas the rpoB^*^ mutation has been shown to compensate for the loss of PBP1 transpeptidase activity (10, 13). *mecA*^+^ rpoB^*^ cells, and those of other highly resistant MRSA strains, are smaller in size and may have a thicker cell wall compared with MSSA strains (13). To represent the *mecA*^+^ rpoB^*^ strain in our model, we use altered parameter values for cell volume (*V_cell_* = 0.60 µm^3^) and cell wall thickness (*t* = 27 nm) (13). Since the structure of the septum is similar between *mecA*^+^ rpoB^*^ and wild-type (13), we assume the same septal-cell wall stiffness ratio (*E_r_* = 1.2) and the same value for turgor pressure, *P* = 2 MPa. All parameter values for the MSSA and MRSA strains are listed in Table 1.

**TABLE 1:** Parameter values used in this work^*a*^

	MSSA (SH1000)	High-resistant MRSA (*mecA*^+^ rpoB^*^)	Low-resistant MRSA (*mecA*^+^)	Reference
Cell volume *V_cell_* (μm^3^)	1.22	0.6	1.30	(13, 31)
Pressurized cell radius (nm)	663	523	677	(13, 31)
Cell wall thickness *t* (nm)	20	27	23	(13)
Poisson’s ratio *ν*	0.499	0.499	0.499	(28)
Turgor pressure *P* (MPa)	2	2	2	(4)
Septum stiffness relative to wall *E_r_*	1.2	1.2	1.2	(25)
Young’s modulus of wall *E* (MPa)	200	200	200	(32)

^*a*^ The cell volume listed is the pressurized volume, *V*_cell_. This is related to the pressurized cell radius ap by $a_{p}=\sqrt[{3}]{\frac{3V_{\text{cell}}}{4\pi }}$. The radius a of an uninflated cell is then calculated from the pressurized radius *a_p_* using $a_{p}=a+Pa^{2}(1-\nu )\;/\;(2Et)$ where *ν* is Poisson’s ratio and *E* is the Young’s modulus of the cell wall material.

**Fig 4:**
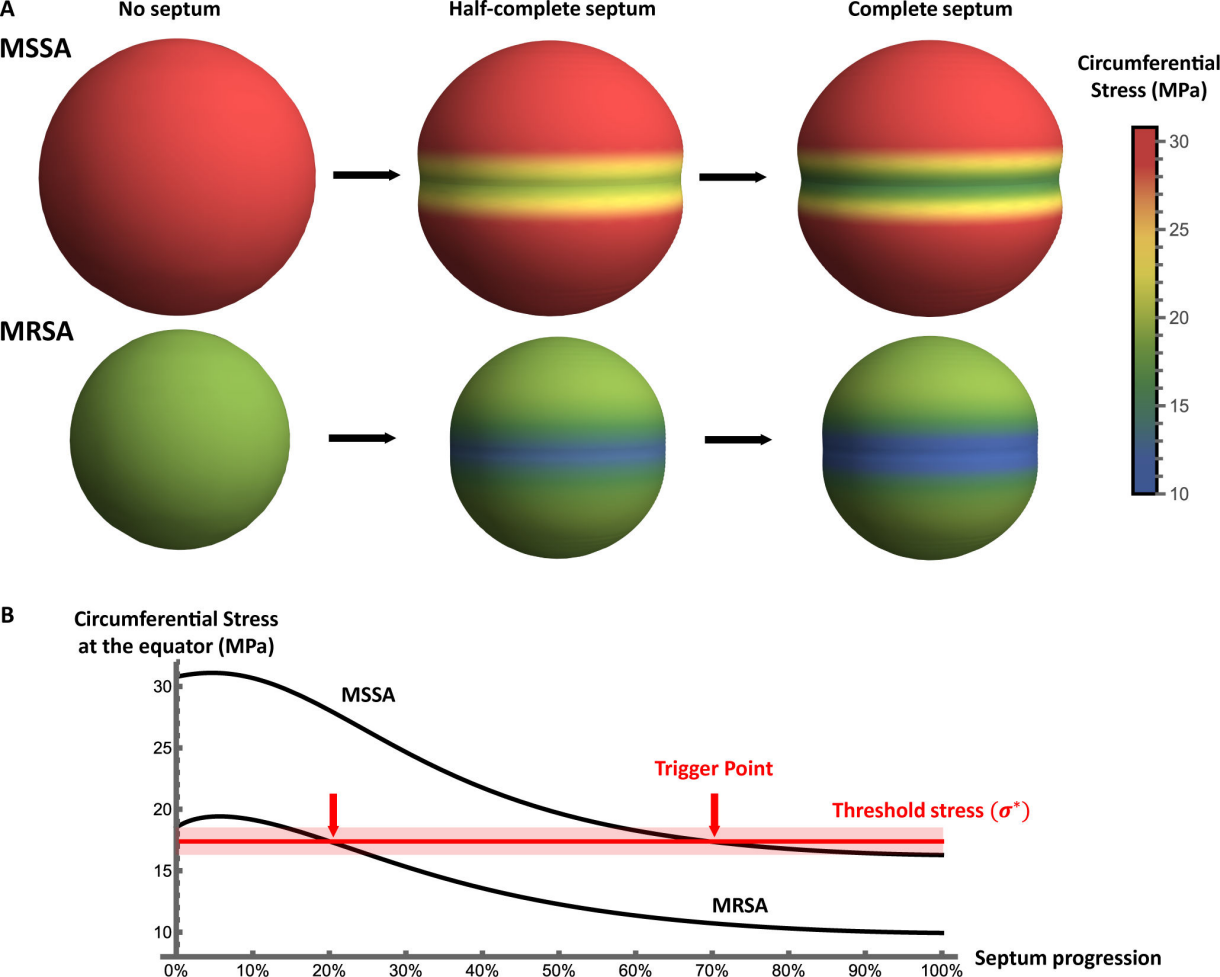
Cell wall stress in an MRSA strain vs MSSA. (**A**) Circumferential mid-plane stress in the cell wall is calculated in phases 1, 2, and 3 of the cell cycle for parameters corresponding to the highly resistant MRSA strain *mecA*^+^ rpoB^*^ (cell volume and thickness: 0.6 µm^3^ and 27 nm), and the corresponding wild-type strain SH1000 (cell volume and thickness: 1.22 µm^3^ and 20 nm). In both cases, *P* is taken to be 2 MPa and *E_r_* is 1.2. Cells of the MRSA strain are smaller with a thicker cell wall; therefore, the cell wall stress is considerably lower in this strain compared to MSSA. (**B**) The calculated circumferential stress at the cell equator as a function of the degree of septum progression for the two strains (septum progression is defined as *a−b/a*, where *a* is the radius and *b* is the aperture size). The threshold stress for triggering autolysin activity (red line) is inferred from the fact that both strains must trigger autolysins, since they can both successfully divide. The arrows show that autolysins are triggered earlier in the cell cycle for the MRSA strain compared with the MSSA wild-type strain.

Calculating the stress distribution in the cell wall for the MRSA strain, we find that the stress is overall much lower than for MSSA (∼18 MPa for MRSA in phase 1 versus ∼30 MPa for MSSA). However, the qualitative pattern of stress through the cell cycle remains similar, with a band of lower circumferential stress developing at the cell equator as the septum forms (Fig. 4).

Bringing together our predictions for the MSSA and MRSA strains, we can estimate the threshold stress $\sigma^{*}$ at which autolysins are activated in the mechanical trigger model. Both the MSSA and MRSA strains can divide (in the absence of antibiotics), implying that both strains successfully trigger autolysin activity. Therefore, the threshold stress must lie within the range of stresses at the equator experienced during the cell cycle for both models (Fig. 4). Examining our model predictions, this implies a value for the threshold stress $\sigma^{*}$ of 17.4 ± 1.1 MPa.

Our model also predicts that the MRSA strain triggers autolysins much earlier in phase 2 compared with the MSSA strain (Fig. 4). For MSSA, the threshold for triggering autolysin activity is reached late in phase 2 (when septum progression, defined as (a-b)/b, where a is cell radius and b is aperture size, is more than 60%). In contrast, for the MRSA strain *mecA*^+^ rpoB^*^, the threshold is reached soon after septum formation starts (when septum progression is between 15% and 25%). We note that up to now, the turgor pressure of MRSA cells relative to MSSA has not been measured; a difference in turgor could alter the picture.

These differences in autolysin triggering time have implications both for cell cycle timing and for the cell’s response to antibiotics, as we discuss later.

### Mechanical trigger model predicts alternative cell fates upon division

The mechanical trigger model also reveals distinct pathways by which *S. aureus* cell division can fail. One failure pathway corresponds to premature cell splitting, that is, the cell splits before the septum is complete, leading to death via lysis at the division site. In the model, this occurs if the relative timing of septum synthesis and wall hydrolysis is such that autolysins complete their task before septum synthesis finishes. Alternatively, division can fail if the cell forms a septum but does not split, that is, it becomes arrested in phase 3. This might lead to stasis, cell clumping, or, if an antibiotic is present that degrades peripheral PG, lysis of the peripheral wall might eventually occur. Our model predicts failure to split if the trigger stress for autolysin activation is not reached, for example, because the circumferential stress is still above the threshold even after completion of the septum (lower blue region) or (depending on the molecular mechanisms involved) if the stress is always lower than the threshold stress (upper blue region).

The existence of distinct division failure pathways is consistent with observations. For example, premature splitting leading to lysis has been reported for MSSA cells in the presence of methicillin (7) and for an MSSA *clpX* mutant grown at 30℃ (8). Failure to split, leading to an increase in the fraction of cells in phase 3, has been reported upon oxacillin treatment for both wild-type cells and for the *clpX* mutant at 30℃ (8). We note that the septum completion signal model, in which autolysis is triggered upon septum completion, does not explain either premature splitting or failure to split after the septum is completed, that is, arrest in phase 3.

**Fig 5:**
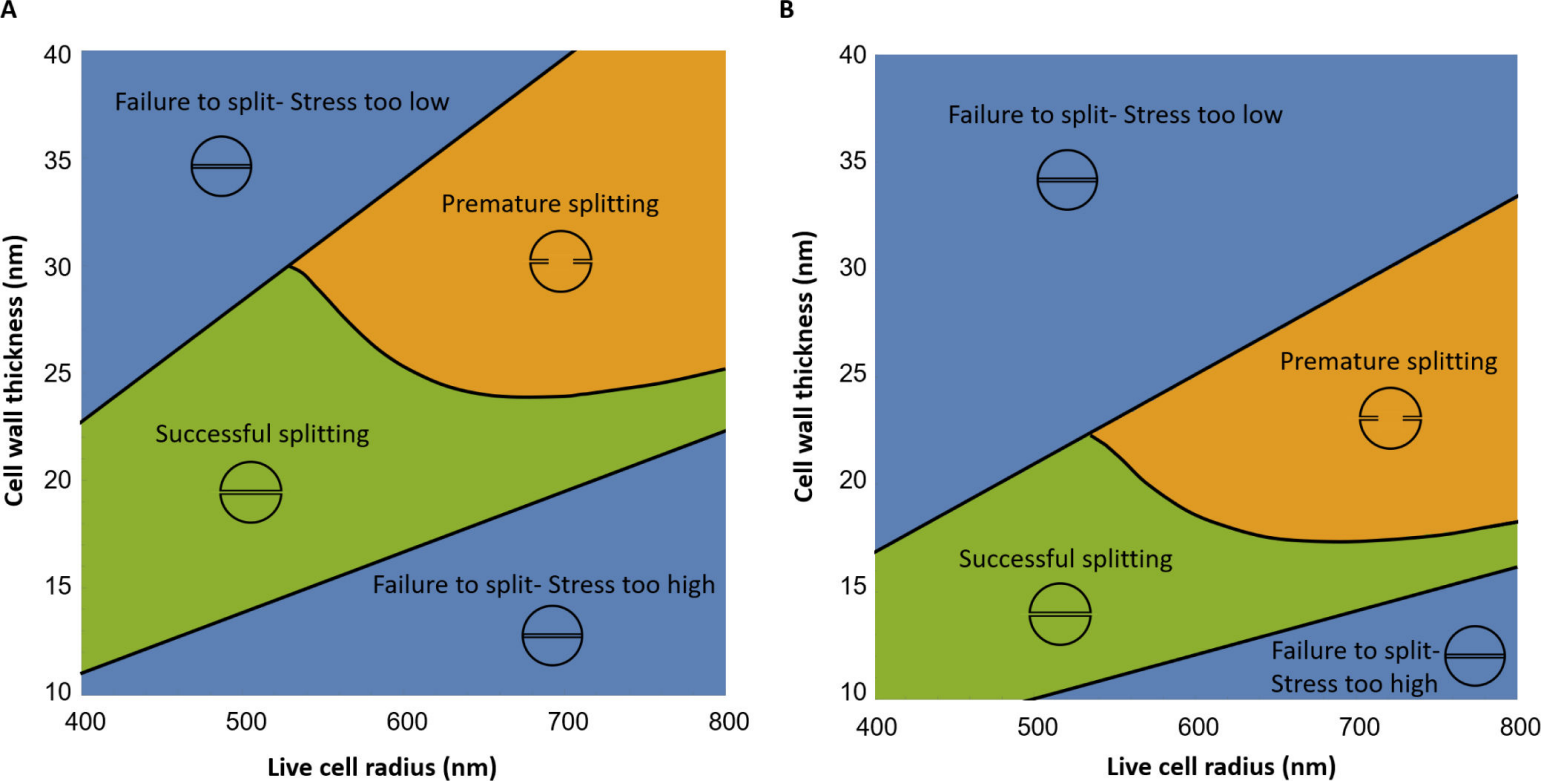
Alternative division fates depend on cell geometry. Cell fate maps showing how the outcome of division is predicted to depend on cell radius and cell wall thickness. In the green region, the model predicts successful division. In the orange region, premature splitting leading to lysis is predicted because wall hydrolysis is completed before septum completion. In the blue region, the model predicts failure to split because the stress threshold for autolysin activation is not reached. The location of the boundary between successful division and premature splitting depends on the relative rate of synthesis and hydrolysis, as explained in the Methods section. Panel (**A**) shows results for turgor pressure *P* = 2 MPa, whereas panel (**B**) shows results for a lower turgor pressure, *P* = 1.5 MPa. In both cases, *E_r_* = 1.2.

In our model, the fate of an *S. aureus* upon division depends on its mechanical and geometric properties as well as the relative rates of PG hydrolysis and synthesis, since these parameters determine the timing and speed of septum synthesis and wall hydrolysis. The role of cell geometry is especially interesting since cells tend to change size and/or wall thickness when they are either challenged by *β*-lactam antibiotics or become resistant to them (7, 13). To probe how cell fate depends on geometry, we plotted a "cell fate map" (Fig. 5A), showing the range of cell radii and cell wall thickness for which (i) the septum is completed before the completion of wall hydrolysis by autolysins, corresponding to successful division (green region in Fig. 5A), (ii) the wall is hydrolyzed before septum completion, corresponding to premature splitting (orange region in Fig. 5A), or (iii) autolysins are not triggered – corresponding to failure to split (blue region Fig. 5A). In these calculations, we assume fixed values for the other model parameters (turgor pressure, septal stiffness relative to wall and ratio of PG synthesis and hydrolysis rates; see Fig. 5 caption). Details of the calculations are given in the Methods section. As anticipated, our results point to cell size and cell wall thickness as key determining factors for cell fate on division. If division failure does occur for small cells, it is in the form of failure to split (e.g., if the cell wall is very thick). In contrast, large cells are, in general, more prone to division failure and can undergo either premature splitting, leading to lysis, or failure to split.

To investigate more systematically how the model parameters influence cell division, we performed a sensitivity analysis to determine the contribution of different parameters to the predicted circumferential stress in phase 1 and phase 2 of the cell cycle (see Supplementary Information, Section VIII). This analysis confirmed the importance of cell size and wall thickness and also revealed a key role for the turgor pressure. Indeed, when plotted for a lower turgor pressure, the cell fate map shifts toward lower values of the cell wall thickness (Fig. 5B). In general, successful division becomes less likely at lower turgor pressure, since the range of parameters for successful division (green region) shrinks. However, lower turgor pressure may be favorable in some cases. For example, a cell that previously failed to split because its mechanical stress was too high could be rescued by decreasing its turgor pressure.

Within an isogenic population of *S. aureus,* cells vary in both cell size and cell wall thickness (13). In the context of the mechanical trigger model, this suggests that individual cells within an isogenic population might experience different division outcomes. Even under conditions where the population as a whole is able to grow, a fraction of cells is predicted to either fail to split or experience premature splitting. Indeed, several reports confirm that individual cells within an *S. aureus* population do show different division outcomes (7, 8).

### Cell size and wall thickness also influence cell cycle timing

**Fig 6:**
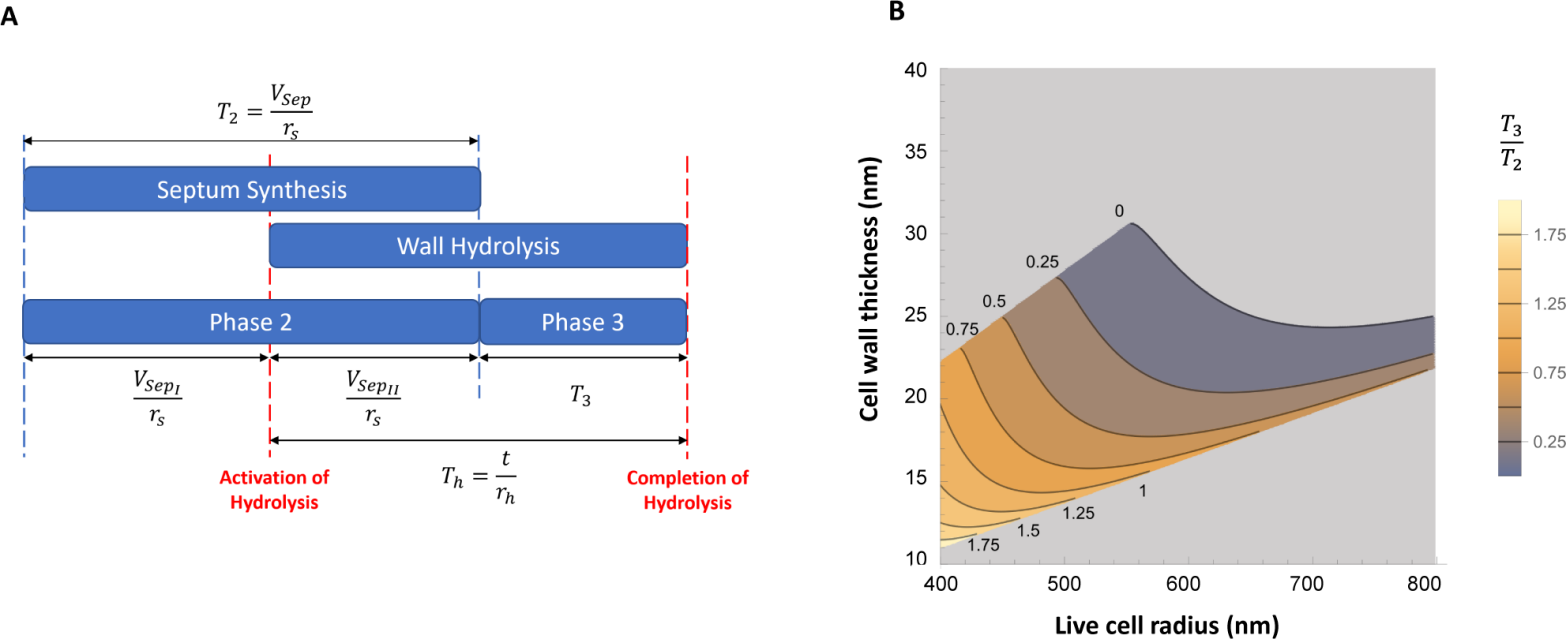
Model implications for cell cycle timing. (**A**) In the mechanical trigger model, the duration of phase 2 corresponds to the time needed to synthesize the septum ($T_{2}=V_{sep}/r_{s}$, where $r_{s}$is the septal synthesis rate and $V_{sep}=2\pi a^{2}t$ is the volume of the two septa, with *a* being cell radius and *t* cell wall thickness). However, since wall hydrolysis starts already in phase 2, the duration of phase 3 depends on both the total time needed to hydrolyze the peripheral ring ($T_{h}=t/r_{h}$ where $r_{h}$ is the PG hydrolysis rate), and the time in phase 2 at which autolysis starts. *T*_*3*_ can be expressed as $T_{3}=T_{h}-V_{{sep}_{II}}/r_{s}$, where $V_{{sep}_{II}}$ is the volume of septum that remains to be synthesized after the autolysin activation. (**B**) Predictions of the mechanical trigger model for the ratio of the duration of phase 3 to phase 2 ($T_{3}/T_{2}$), as a function of the cell radius and cell wall thickness. In the light gray region, the model does not predict successful division, and timing is not relevant. In these calculations, the septum relative stiffness is taken to be *E_r_* = 1.2 and the turgor pressure *P* = 2 MPa.

For cells that undergo a successful cell cycle, the mechanical trigger model also makes predictions about cell cycle timing. Specifically, the relative time that the cell spends in phase 2 and phase 3 is predicted to depend on the mechanical and geometrical properties of the cell, as well as the relative rates of septal synthesis and peripheral ring hydrolysis (Fig. 6).

In this model, the duration of phase 2 (*T_2_*) is the time taken to synthesize the septum, which depends on cell radius, septum thickness, and the rate of synthesis of septal PG ($T_{2}=V_{sep}/r_{s}$, where $r_{s}$ is the septal synthesis rate and $V_{sep}=2\pi a^{2}t$ is the volume of the two septa). The duration of phase 3 (*T_3_*) is, however, more complex, since it is determined by the amount of time needed to complete peripheral ring hydrolysis, after septal synthesis finishes (Fig. 6A). This involves an interplay between two factors: the total time *T_h_* needed to hydrolyze the peripheral ring, that depends on autolysin activity and wall thickness (here we assume that $T_{h}=t/r_{h}$, where $r_{h}$ is the PG hydrolysis rate; see the methods section), and the time in phase 2 at which autolysis starts, which depends on mechanical stress at the cell equator and hence on all the geometrical and mechanical properties of the cell. The duration of phase 3 can be written as $T_{3}=T_{h}-V_{{sep}_{II}}/r_{s}$, where $V_{{sep}_{II}}$ is the volume of septum that remains to be synthesized after the activation of hydrolases (Fig. 6A); this depends on the septum aperture size at triggering, which can be calculated with the mechanical trigger model (see Methods). Therefore, the relative time that the cell spends in phase 2 and phase 3 can be predicted:


(3) $$\frac{T_{3}}{T_{2}}=\frac{\left(r_{s}/r_{h}\right)t-V_{{sep}_{II}}}{V_{sep}}.$$

For more details of these calculations, see the Methods section.

The model predicts that *S. aureus* strains with different geometrical or mechanical properties, or with different PBP or autolysin activity, will show different cell cycle timing. Assuming a fixed relative rate of septal synthesis to wall hydrolysis, we can predict how $T_{3}/T_{2}$ depends on cell radius and cell wall thickness (Fig. 6B). In general, increasing either cell radius or cell wall thickness tends to decrease the duration of phase 3 relative to phase 2. These predictions contrast with those of the septum completion signal model. In that model, the duration of phase 3 would simply equal the time needed to hydrolyze the peripheral ring; therefore, *T_3_* would be predicted to depend only on autolysin activity and wall thickness and to be independent of other properties of the cell, such as cell radius, septum synthesis rate, wall stiffness, or turgor pressure.

### Arrest of septal synthesis causes alternative, geometry-dependent fates

In the mechanical trigger model, an irreversible commitment to scission occurs during phase 2 when the critical stress threshold is reached, triggering autolysin activity. If septal synthesis becomes arrested (for example, upon exposure to antibiotics), the fate of the cell differs depending on the time at which the arrest happens. If septal synthesis arrests before the stress threshold is reached, the model predicts that autolysins will not be activated and the cell will not split, but instead, it becomes arrested in phase 2. However, if septal synthesis is arrested after the stress threshold is reached, autolysin activity is already underway, and the cell will eventually split with an incomplete septum.

**Fig 7:**
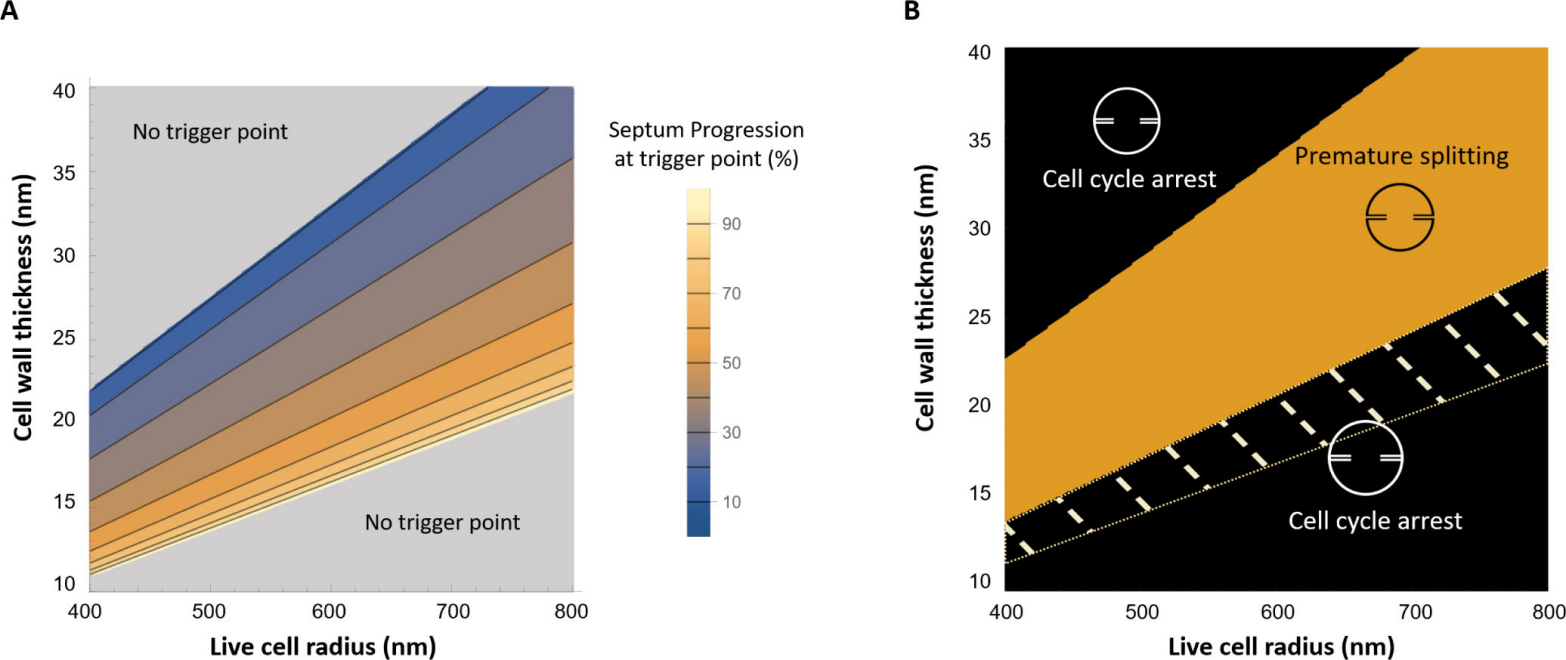
Predicted cell fate upon arrest of septal synthesis. (**A**) The time in phase 2 at which the cell commits to scission depends on the geometrical and mechanical properties of the cell. The colors indicate the extent of septum progression (during phase 2) at which the stress threshold is reached for triggering of autolysin activity. Cells that are smaller or have a thicker cell wall tend to trigger autolysin activity earlier in phase 2. (**B**) Prediction for cell fate assuming that septum formation stalls at 50% progression (defined as (*a − b)/a* where *a* is the radius and *b* is the septum aperture size). If stalling occurs after autolysins are triggered, the cell undergoes premature splitting (orange region). If autolysins are not triggered, the cell is arrested in phase 2 (black regions). For cells in the shaded region, the septum stalls before the hydrolase trigger event; for cells in the black but not shaded region, hydrolases are never triggered even without septum stalling (see Fig. 5). In these calculations, septum relative stiffness is taken to be *E_r_* = 1.2 and turgor pressure *P* = 2 MPa.

The commitment point during phase 2 (i.e., the degree of septum progression at which autolysins are triggered) depends on the cell wall stress and hence on the geometrical and mechanical properties of the cell (Fig. 7A). Cells that are smaller or have a thicker cell wall tend to trigger autolysin activity earlier in phase 2, whereas cells that are larger or have a thinner cell wall tend to trigger later. Therefore, the cell fate upon arrest of septal synthesis is predicted to depend on cell geometry. For example, stalling of septum synthesis at 50% progression is predicted to lead to scission (with an incomplete septum) for smaller or thicker cells that have already triggered autolysin activity, whereas larger or thinner cells are predicted to arrest in phase 2 without splitting, since they have not yet triggered autolysin activity.

Arrest of septal synthesis has been linked with premature cell scission in several contexts. The antibiotics methicillin and vancomycin arrest both septal and peripheral PG synthesis: methicillin exposure can cause lysis via premature scission (as we discuss in more detail below), whereas vancomycin, which inhibits the activity of Atl, does not (7) (an *atl* mutant also shows reduced death rate in the presence of methicillin but not vancomycin). This is consistent with our model, showing that premature splitting requires the combined effect of septum stalling and the activity of hydrolases. In a different study, contrasting outcomes were obtained for two MRSA strains exposed to the antibiotic daptomycin (9). Cells of strain SADR-1, which became arrested at an early stage of septum progression, could not continue peripheral synthesis and eventually lysed via premature splitting, whereas SADR-2 cells (which have mutations in *rpoB* and *clpP*) arrested before septum initiation, continued uniform peripheral growth, and resumed division when daptomycin was removed (9). Further evidence that arrest of septal synthesis can lead to premature splitting comes from a study of a strain lacking the ClpX chaperone; in this strain, when grown at 30℃, septum synthesis occasionally stalls at an early stage, which leads to premature splitting (8). At 37℃, septum stalling and subsequent premature splitting is not frequent, although autolysin expression levels are similar at both temperatures (8). Also hinting at a link between septum formation and autolysin activity, treatment of *S. aureus* with the antibiotic penicillin is associated with both defects in septum formation (33) and autolysin-dependent cell lysis (22).

### The model can rationalize the complex effects of methicillin

The effects of the *β*-lactam antibiotic methicillin on *S. aureus* cells have been well characterized (7, 13). For MSSA cells, high-concentration methicillin exposure (above the MIC) halts both peripheral and septal PG synthesis, whereas PG hydrolysis remains uninhibited; the resulting imbalance between synthesis and hydrolysis leads to death (7). Cells die after around 120 min of exposure via one of two pathways: "peripheral death," that is, cell lysis due to failure of the peripheral cell wall, or "septum-associated death," that is, premature cell scission leading to lysis at the division site (7). Prior to death, the cells increase in size, and atomic force microscopy shows that the peripheral cell wall becomes thinner with perforating holes, whereas the septum loses its concentric ring structure (13), suggesting that it has become softer (25). Around 40% of methicillin-exposed MSSA cells experience plasmolysis, that is, retraction of the cell membrane from the wall, suggesting decreased turgor pressure (7). In some cases, plasmolysis is associated with death via premature cell scission (7).

It is clear that, in addition to arresting PG synthesis, methicillin exposure impacts the geometrical and mechanical properties of the cell, including size, peripheral and septal PG stiffness, and turgor pressure, which are relevant in the mechanical trigger model. For an uninhibited MSSA cell, the model predicts that autolysins are activated late in phase 2, when the septum is more than 60% complete (as discussed earlier). Assuming that methicillin immediately stalls septum synthesis, the model predicts different fates for cells that have or have not reached this commitment point at the time of methicillin addition. Cells in phase 1 or early in phase 2 have not yet activated the scission autolysins; these cells are predicted to stall in phase 1 or 2. Due to the action of methicillin, the wall hydrolysis (away from the division site) and synthesis are imbalanced, the peripheral wall weakens, and the cell is expected to eventually undergo peripheral death.

In contrast, cells that are in late phase 2 have already triggered the scission autolysins at the time of methicillin addition. For these cells, methicillin-associated stalling of septal synthesis is predicted to lead to premature scission and hence septum-associated death. Interestingly, the loss of turgor pressure that is associated with plasmolysis may trigger autolysin activation even in cells that have not yet reached the normal trigger point, leading to plasmolysis-related septum-associated death even for cells that were early in phase 2 at the time of methicillin addition. This prediction is consistent with the observed link between plasmolysis and septum-associated death (7). Finally, if a cell is in phase 3 when methicillin is added, the model predicts that the cell can complete scission, although the daughter cells will eventually undergo peripheral death.

### The model points to a key role for cell geometry in high-level MRSA

MRSA strains of *S. aureus* have an alternative transpeptidase, PBP2a (encoded by the *mecA* gene), which has a low affinity for *β*-lactam antibiotics and compensates for the loss of PBP2 activity. In an SH1000 strain background, acquisition of *mecA* has little effect on cell size and wall thickness compared with the MSSA wild-type strain (31). The *mecA*^+^ strain is able to synthesize peripheral and septal PG in the presence of methicillin, but this strain shows only low-level methicillin resistance (13). High-level resistance is conferred by the acquisition of an additional potentiator mutation, such as rpoB^*^ (34). As discussed earlier, cells of the *mecA*^+^ rpoB^*^ strain are smaller than those of MSSA and have a thicker cell wall.

**Fig 8:**
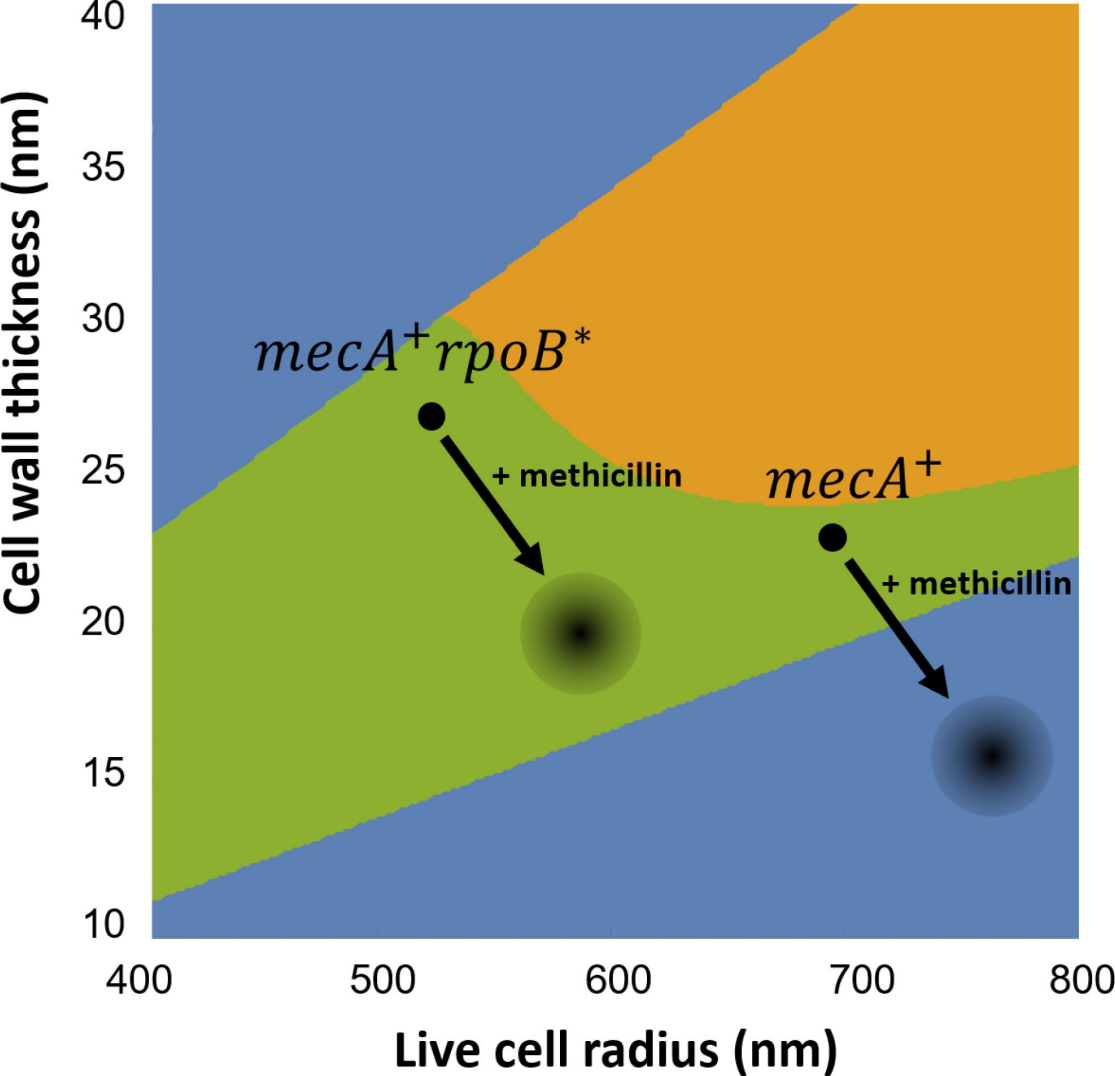
Model prediction for the fates of low-level and high-level MRSA cells in the presence of methicillin. The cell fate map shows the location of the low-level MRSA strain *mecA*^+^ (cell volume and thickness: 1.30 µm^3^ and 23 nm) and the high-level MRSA strain *mecA*^+^ rpoB^*^ (cell volume and thickness: 0.6 µm^3^ and 27 nm) in the absence of methicillin. Both strains are predicted to divide successfully (green region of the cell fate map). In the presence of methicillin, cells of both strains become larger and the cell wall becomes thinner (13). These geometrical changes (shown by arrows) cause the low-level MRSA strain (*mecA*^+^) to move from the green region (corresponding to successful division) to the blue region, corresponding to failure to initiate splitting and arrest in phase 3, presumably leading to eventual lysis by mechanical failure as the cell wall continues to weaken. In contrast, the high-level MRSA (*mecA*^+^ rpoB^*^) cells remain in the green region, implying that they continue to divide successfully in the presence of sub-MIC concentration of methicillin. In these calculations *E_r_* is taken to be 1.2 and *P* = 2 MPa (Table 1).

The mechanical trigger model shows how the difference in geometry between the *mecA*^+^ and *mecA*^+^ rpoB^*^ strains could explain their differing resistance levels. In the absence of methicillin, both strains lie in the region of the cell fate map that corresponds to successful division, although they lie in different places in the map due to their different geometries (Fig. 8). In the presence of methicillin, cells of both strains increase in size and decrease in cell wall thickness (13). Assuming that the turgor pressure and the wall stiffness remain unchanged, geometrical changes upon addition of methicillin move the location of these cells on the fate map in the direction of the bottom right corner (the arrows on Fig. 8). The model predicts that cells of the high-level MRSA *mecA*^+^ rpoB^*^ strain retain the ability to divide successfully in the presence of methicillin (i.e., they remain in the green region of the cell fate map); however, the addition of methicillin moves the *mecA*^+^ cells from the green region to the blue region, where cell scission fails and cells become arrested in phase 3 (Fig. 8). Since methicillin weakens the peripheral wall, we speculate that these cells may eventually undergo peripheral death. Therefore, smaller cells with a thicker wall retain their ability to divide successfully in the presence of high concentrations of methicillin, but larger, thinner cells do not. Essentially, methicillin causes cells to become larger and thinner, which is not compatible with successful division for cells that initially have a normal geometry. However, by being initially smaller and thicker, high-level MRSA cells can offset this effect, remaining within the geometric range for successful division even in the presence of methicillin. Methicillin is also expected to decrease septum stiffness by removing the septal ring structure (13, 25); however, our calculations suggest that the effect of septum stiffness is generally less important than that of cell geometry (see Supplementary Information, Section VII).

Interestingly, our model suggests that when an MRSA strain is exposed to antibiotic close to or above its MIC, it will typically show failure to split, rather than premature splitting (Fig. 8). Consistent with this, cells of the MRSA strain USA300 have been reported to fail to split in the presence of the *β*-lactam antibiotic oxacillin, leading to an increase in the number of cells in phase 3 (although we note that Sle1 expression is also decreased in the presence of oxacillin) (17).

## DISCUSSION

Bacterial cell division is an intricate process that coordinates mechanical forces with the activities of multiple biochemical players. In *S. aureus*, cell division involves changes in mechanical stress in the PG cell wall and the action of PG synthase and hydrolase enzymes that remodel the wall; however, it is not well understood how division is controlled, particularly the final step in which the cell splits into two daughter cells. In this work, we presented a model for control of cell scission in *S. aureus*. We first used thin shell mechanics to predict the changing pattern of mechanical stress in the PG cell wall through the cell cycle, finding that the circumferential stress decreases markedly at the equator of the peripheral wall, close to the division site, as the septum is formed. Building on the long-standing concept of stress regulation of PG hydrolases (20), we hypothesized that this local decrease in stress might trigger the activity of the autolysins (Atl and/or Sle1) that cleave the peripheral ring, leading to cell scission. Our model’s strength is its conceptual simplicity and the fact that it links cell geometry with division fate.

In this mechanical trigger model, commitment to scission occurs during phase 2 of the cell cycle, before the septum is complete, at a point that depends on geometrical and mechanical properties of the cell, namely cell size, PG wall thickness, turgor pressure, and relative stiffness of the septum and the peripheral wall, as well as the relative rates of PG synthesis and hydrolysis. The mechanical trigger model suggests three alternative outcomes for cell division: (i) successful division, (ii) premature scission with an incomplete septum, or (iii) division arrest due to failure to split. Which outcome occurs depends on cell geometry, mechanics, and biochemistry, which can be altered by mutations or by environmental conditions such as osmotic or temperature changes or the presence of antibiotics; these factors also influence cell cycle timing. Live-cell microscopy experiments that track the division outcomes of individual cells (e.g., using microfluidic technologies), under varying osmotic and temperature conditions and for mutants with different geometric and mechanical properties, will be important in testing these predictions (35). The mechanical trigger model shows how cell size and cell wall thickness are major determinants in the outcome of cell division. It can also explain diverse observations, including that premature scission occurs under varied conditions (7–9), that methicillin treatment can cause death by either of two pathways (7), and that cells of high-level MRSA strains are typically small with thick cell walls (13).

In the mechanical trigger model, the cell wall stress, and hence the outcome of division, emerges from the integration of multiple factors. This implies that some properties of the cell can compensate for others. For example, the model predicts that a small *S. aureus* cell with a thick wall might fail to split, but adding *β*-lactam antibiotics, which increase cell size, might help it to divide successfully. Along similar lines, if a strain grows slowly because it spends a long time in phase 3 of the cell cycle, increasing the expression level of autolysins could promote growth. This makes it hard to make simple general predictions, since the model suggests that some properties of a cell (e.g., turgor pressure) may compensate for others (e.g., cell size) in determining division outcome. This potential for compensatory effects may help explain apparently counterintuitive observations that antibiotics and/or autolysins can promote growth in some cases (8, 17).

Our model may help explain the complex response of *S. aureus* strains to *β*-lactam antibiotics, in particular, methicillin. For MSSA cells, the model suggests that septum-associated death occurs in cells that are in phase 2 at the time of methicillin exposure and have triggered autolysins (either because the trigger point has been passed or because of subsequent loss of turgor pressure, associated with plasmolysis). In contrast, peripheral death is predicted for cells that were in phase 1 or early phase 2 (having not yet triggered autolysins) at the time of methicillin exposure. Cells that were in phase 3 are predicted to continue division, with the daughters then experiencing peripheral death. It will be very interesting to test these predictions in live-cell microscopy experiments while also measuring how cell size and cell wall thickness change in time after methicillin exposure, for both sub-inhibitory and killing concentrations. Interpreting such measurements would also require a dynamical version of the model that accounts in more detail for PG synthesis and hydrolysis around the division site and takes into account changes in parameters over time (both during the cell cycle and after methicillin addition). Such a model could also include adaptation mechanisms that can compensate for the inability to split, for example, by changing the turgor pressure.

Pioneering previous work has also implicated mechanical stress in *S. aureus* cell scission (5). In that work, the same thin shell mechanics approach was used to calculate the stress distribution in the cell wall during the cell cycle, but it was assumed that the peripheral ring of PG around the division site does not grow with the rest of the cell, implying a local band of high stress in the peripheral ring. The local high stress was proposed to cause mechanical failure, leading to perforations in the peripheral ring, which is followed by mechanical crack propagation leading to scission. The role of hydrolases remains ambiguous in this picture. Here, we do not focus on crack propagation but rather on the cause of the initial perforations in the peripheral ring. Our model suggests that these perforations may be an outcome of the interplay between mechanical and biochemical players. Specifically, we propose that the decrease in stress in the cell wall close to the division site, which is caused by septum formation, could trigger autolysin activity that eventually leads to holes in the PG. The formation of these holes may be accompanied by crack propagation, as proposed in (5), although this is not the focus of our model. Crack propagation, if it occurs, is likely to be purely mechanical, since the timescale of crack dynamics is fast compared to that of hydrolase activity. The mechanical trigger model proposed in this work brings together mechanical and biochemical factors and allows us to predict the effect of different geometrical and mechanical properties of the cell on the outcome of division and the timing of the cell cycle.

Our model is built on the hypothesis that a local reduction in circumferential mechanical stress around the division site can trigger the activity of autolysins. Although the concept of stress regulation of hydrolases has long been discussed (20), it remains unclear how such regulation might work mechanistically. Several studies on different hydrolases have shown that a conformational switch controls activity (36, 37). We speculate that cell wall mechanical stress might alter the relative stability of active and inactive hydrolase conformations, for example, via a Monod-Wyman-Changeux mechanism (38). It is also possible that activation happens through a cascade of events involving multiple molecular players. Notably, in our cell fate map (Fig. 5), we have assumed that autolysins activate only when they encounter a specific value of the circumferential mechanical stress, that is, they remain inactive if the stress is always higher than the threshold, or if it is always lower than the threshold (corresponding to the bottom right and top left blue regions in Figure 5, respectively). Our model still holds if, instead, autolysins are active at any stress that is below the threshold. In that case, the top left region of the cell fate map would not correspond to failure to split but rather to a situation where cells start to hydrolyze the peripheral ring as soon as autolysins are localized at the division site, since in these cells, the wall stress in phase one is already lower than the threshold value.

In this work, we have assumed a single value of the stress threshold for autolysis activation across multiple *S. aureus* strains (the value of the stress threshold was set by requiring successful division for both MSSA and MRSA). This assumption arises from our speculation that the stress threshold is an intrinsic property of the hydrolases (Atl/Sle1). The use of a single threshold value provides a simple framework that allows us to focus on the effect of key physical properties of the cell (size and wall thickness) on the division outcome. We have also assumed that the value of the turgor pressure does not differ between the MSSA and MRSA strains. If the turgor pressure or the stress threshold for hydrolase activation were strain-dependent, this would, of course, change our quantitative predictions for strain-to-strain differential outcomes—this is one reason why we have limited our study to mainly qualitative predictions. However, even within an isogenic population of cells, where stress threshold and turgor pressure can presumably be assumed to be constant, cell size and cell wall thickness vary among cells, leading to different predicted cell division outcomes for different cells in the population.

Although our model qualitatively explains a range of experimental phenotypes, a quantitative comparison of the model predictions with experimental observations in the literature is not straightforward since few studies combine wall thickness and cell size measurements with those of single cell fates and cell cycle timing. Also, the central prediction of our model, that peripheral ring hydrolysis starts during phase 2 at a time that depends on cell geometry and mechanics, is unfortunately not easy to test experimentally. However, advanced techniques are increasingly being used to study *S. aureus*; atomic force microscopy (AFM) is revealing the detailed structure and mechanics of the cell wall with fine spatial resolution, in some cases for live dividing cells, while microfluidics provides the potential to monitor the fates of single cells upon dynamic exposure to antibiotics or environmental changes. An interesting avenue would be to monitor, using AFM or electron microscopy, the timing and patterning of the appearance of holes around the division site, which provides evidence of autolysin activity at the start of scission, for different strains and conditions. Advanced methods such as cryo FIB-SEM might allow the simultaneous imaging of septum progression and the start of peripheral ring hydrolysis. Such methods could also be applied in the presence of *β*-lactam antibiotics to better understand how peripheral death occurs, for example, to compare measurements of the septum aperture size at the time of scission to model predictions. It would also be very interesting to connect the geometrical and mechanical properties of different *S. aureus* strains with their cell cycle timing. For example, the dependence of the duration of phase 3 on cell properties is a key prediction of the mechanical trigger model that differs from the septum completion trigger model. Also interesting here would be to link the cell cycle timing and division outcomes of individual cells to the population-level outcomes that are measured in typical microbiological assays. To predict population-level outcomes, the mechanical trigger model should be integrated with equations for the population dynamics of subpopulations of cells in the different phases of the cell cycle (39).

A promising experimental approach to test our model predictions would be changing the osmolarity of the medium; hence, the turgor pressure difference between the cell interior and exterior. However, osmolarity experiments have limitations that make direct comparison with our model challenging. Changing external osmolarity is a transient process that triggers short-term responses, different from steady-state behavior. Additionally, osmoregulation (40) allows the cells to adapt to the new osmotic conditions. Lowering medium osmolarity has been shown to increase the number of “popping” events (5). This increase, however, is mainly observed during the transition phase, and after some seconds, the number of popping events decreases again. From our model’s perspective, depending on the cell size and wall thickness, either lower or higher turgor pressure may be favorable in different cases. However, lower turgor pressure reduces the likelihood of successful splitting (Fig. 5). In order to make direct comparisons, we would also need measurements of the cell size and wall thickness, which are currently not available for the osmolarity experiments reported in the literature.

It is important to note that our model makes a clear distinction between the expression levels of autolysins (Atl/Sle1) and their activity levels. In the model, autolysins are assumed to be expressed throughout the cell cycle, localized to the division site, but are only activated at the threshold trigger stress. Their expression level is predicted to alter the rate of hydrolysis in the model, moving the boundary between successful division and premature splitting in the fate map, but not the overall picture. Interestingly, the abundance of the cell wall hydrolases does not always correlate with the cell’s ability to split (41). Although several studies show how autolysin expression levels change under different mutations and conditions (17, 19, 41), direct measurement of their activity is challenging (23). However, methods such as activity-based protein profiling might provide an interesting direction for future research (42). Also, the relative role of autolysins Sle1 and Atl needs to be clarified, as some studies suggest that Sle1 is the main hydrolase responsible for cleaving the peripheral ring while Atl cleaves the inner bonds between the two septa (17).

Finally, our calculations also point to an interesting pattern of mechanical stress that develops in the incomplete septum during phase 2 of the cell cycle: the tangential stress is higher in the inner part of the septum (Supplementary Information, Section III). The implications of this septal stress patterning are currently unclear, but we speculate that PG synthesis might follow the direction of higher mechanical stress. This might be in line with experimental observations that the growing tip of the septum is the most active site of PG assembly (14), and PG forms rings on the septum (26).

In *S. aureus*, septum synthesis needs to be coordinated with cell scission to avoid premature splitting leading to cell death. Here, we showed that a simple mechanical trigger model, in which autolysins are activated by local stress reduction at the division site, caused by septum formation, can explain a number of apparently complex and disparate experimental observations. This study suggests a central role for mechanical stress regulation of PG hydrolases in cell division control and may also be relevant for other gram-positive bacteria. Ultimately, a better understanding of cell division control in *S. aureus* and other bacteria could help develop new strategies against bacterial infection.

## MATERIALS AND METHODS

### Calculation of circumferential stress in the cell wall through the cell cycle

We use a solid elastic model to predict the pattern of mechanical stress in the cell wall during the *S. aureus* cell cycle (Fig. 1). The solid elastic model means we assume that the cell wall retains its shape upon depressurization (loss of turgor) and the deformations of the wall that occur due to pressurization are within the elastic range. In phase 1, the cell wall is modeled as a pressurized spherical thin shell. The shell is assumed to be homogeneous and isotropic, with a thickness that is small compared with its radius. The turgor pressure *P* inflates the spherical shell, stretching the wall and hence generating stress. Due to the spherical symmetry, the stresses in the longitudinal and circumferential directions are equal ($\sigma_{l}=\sigma_{h}=\sigma$) during phase 1. At equilibrium, the force generated by the turgor pressure is balanced by the in-plane forces within the wall. Computing the forces acting on a plane through the center of the sphere (Fig. S1A), force balance implies that $P\pi a^{2}=2\pi at\sigma$; this leads directly to Eq. (1).

In phase 2, the partially formed septum prevents the shell from expanding freely when inflated by the turgor pressure, creating local bending of the wall in the vicinity of the septum. To compute the stress distribution, we model the cell as two hemispheres, each of which is connected to a disc with an aperture, representing the incomplete septum (Fig. 1C). Pressurization of the cell generates a force *T* and bending moment *M_α_* at the boundary between the hemisphere and the disc (Fig. 1C); by computing *T* and *M_α_*, we can obtain the distribution of mechanical stress in the midplane of the cell wall, as described in detail in the Supplementary Information, Section I. Since the spherical symmetry is broken by the formation of the septum, the stresses in the longitudinal and circumferential directions are no longer equal. The longitudinal stress ($\sigma_{l}$) remains the same as in phase 1, but the circumferential stress ($\sigma_{h}$) becomes


(4) $$\sigma_{h}\left(phase\;2\right)=\frac{Pa}{2t}-\frac{T\lambda }{t}e^{-\lambda \psi }\left(\sin\lambda \psi +\cos\lambda \psi \right)$$

where ψ denotes the angle from the equator to the point on the surface where the stress is calculated and $\lambda =\sqrt[{4}]{3\left(1-\nu^{2}\right){\left(\frac{a}{t}\right)}^{2}}$, where ν is Poisson’s ratio. Evaluating eq. 4 at the equator (ψ =0) leads to eq. 2. If we assume that the thickness *t_s_* of the septum increases in proportion to its radial extent, such that $t_{s}=t(a-b)/a$, where *b* is the aperture size (see Supplementary Information, Section IV), the boundary force *T* in eq. 4 (and eq. 2) is equal to


(5) $$T=\frac{\frac{P_{a}^{2}\left(1-\nu \right)}{2t}E_{r}-\frac{2{Pab}^{2}}{a^{2}-b^{2}}}{\left(1-v\right)\frac{a^{4}}{t\left(a-b\right)\left(a^{2}-b^{2}\right)}+\left(1-v\right)\frac{a^{2}b^{2}}{t\left(a-b\right)\left(a^{2}-b^{2}\right)}+\frac{a\lambda E_{r}}{t}}$$

where *E_r_* is the relative stiffness of the septum compared with the peripheral wall. eq. 5 is derived in the Supplementary Information, Section I. eqs. 4 and 5 show that the pattern of stress in phase 2 depends on the septum aperture size b, which decreases as the septum is formed. In phase 3, eqs. 4 and 5 still hold, but since the septal disc is complete, the aperture size b is zero in Eq. 5.

### Septal aperture size at which autolysin activity is triggered

In the mechanical trigger model, we assume that the autolysins that mediate cell scission become activated when the circumferential stress at the equator becomes equal to the threshold stress $\sigma^{*}$. The aperture size *b_t_* at which autolysin activity is triggered can be found by equating the circumferential stress at the equator (eq. 2) with the threshold stress $\sigma^{*}$:


(6) $$\frac{Pa}{2t}-\frac{\lambda }{t}\left(\frac{\frac{P_{a}^{2}\left(1-\nu \right)}{2t}E_{r}-\frac{2{Pab}^{2}}{a^{2}-b^{2}}}{\left(1-v\right)\frac{a^{4}}{t\left(a-b\right)\left(a^{2}-b^{2}\right)}+\left(1+v\right)\frac{a^{2}b^{2}}{t\left(a-b\right)\left(a^{2}-b^{2}\right)}+\frac{a\lambda E_{r}}{t}}\right)=\sigma^{*}$$

If eq. 6 has no solution, the circumferential stress at the equator is never equal to the threshold stress and the autolysins are not activated. This implies that the cell fails to split.

### Ratio of time spent in phase 3 vs. phase 2 of the cell cycle

We assume that septal material is synthesized at a constant rate, so that the duration $T_{2}$ of phase 2 of the cell cycle can be expressed as $T_{2}=V_{sep}/r_{s}$ where $V_{sep}=2\pi a^{2}t$ is the volume of the complete septum and $r_{s}$ is the rate (volume per time) at which septal material is synthesized.

In the mechanical trigger model, autolysins are triggered at some point during phase 2, when the circumferential stress reaches the trigger threshold. We can divide the septal volume into two parts: the part $V_{{sep}_{I}}$ that is synthesized before autolysins are triggered, and the part $V_{{sep}_{II}}$ that is synthesized after the autolysins are triggered (see Fig. 6). Correspondingly, the duration of phase 2 can be split into the times before and after the trigger ($T_{2_{I}}$ and $T_{2_{II}}$, respectively): $T_{2}=T_{2_{I}}+T_{2_{II}}=V_{{sep}_{I}}/r_{s}+V_{{sep}_{II}}/r_{s}$. Considering the geometry of the incomplete septum, we find that ${V_{{sep}_{II}}=2\pi [a}^{2}t-\left(a^{2}-b_{t}^{2}\right)t_{s_{t}}]$, where $t_{s_{t}}$ is the thickness of the septum at the time of triggering ($t_{s_{t}}=t(a-b_{t})/a$; see Supplementary Information, Section IV).

Next, we consider the time $T_{h}$ that is required for autolysins to cleave the peripheral ring and cause scission. Inspired by previous work suggesting that crack propagation is relevant for scission (5), we suppose that the autolysins do not have to digest the entire volume of the peripheral ring, but rather should penetrate its width. Therefore, we suppose that $T_{h}=t/r_{h}$ where t is the thickness of the cell wall and rh is the rate at which the peripheral ring thickness is reduced by autolysins (in units of distance/time). The time *T_h_* can be split into the part that occurs during phase 2, before the septum is complete, and the part that occurs during phase 3, after septum completion (see Fig. 6): ${T_{h}=T}_{2_{I}}+T_{3}$. Using our previous results, this can be written as $t/r_{h}=V_{{sep}_{II}}/r_{s}+\left(T_{3}/T_{2}\right)\left(V_{sep}/r_{s}\;\right)$, which can be rearranged to predict the relative duration of phase 3 to phase 2: $T_{3}/T_{2}\;=\left(\left(r_{s}/r_{h}\right)t-V_{{sep}_{II}}\right)/V_{sep}$ (eq. 3). If the predicted $T_{3}/T_{2}$is negative, the model predicts premature splitting.

### Parameter values

Table 1 lists the parameter values used in this work. The calculations reported in Figure 6 for the relative duration of phases 2 and 3 of the cell cycle ($T_{3}/T_{2}$) require an additional parameter value $r_{s}/r_{h}$, the relative rates of synthesis of the septum (in volume/time) versus hydrolysis of the peripheral ring (in length/time). We also use $T_{3}/T_{2}$ in the calculation of the cell fate maps in Figures 5 and 8 (see below). For an MSSA strain, previous work reports that $T_{3}/T_{2}=1/3$, based on counting the number of cells in phases 2 and 3 in fluorescence microscopy images (8). Inserting this value into eq. 3 and using our MSSA parameter set (Table 1), we infer a value for $r_{s}/r_{h}$ for MSSA. Since the relative activities of cell wall synthases and hydrolases are tightly regulated (19), we assume the same value for $r_{s}/r_{h}$ for other strains and conditions, and use this to infer $T_{3}/T_{2}$.

### Plotting cell fate maps

To obtain the cell fate maps in Figures 5 and 8, we systematically vary the pressurized cell radius between 400 and 800 nm and the cell wall thickness between 10 and 40 nm. From the pressurized radius, we obtain the unpressurized radius *a* as input to the stress calculation, as described in Table 1. We then calculate the circumferential stress at the equator during the cell cycle as described above. If the stress is never equal to the threshold stress $\sigma^{*}$, the mechanical trigger model predicts that autolysins are not triggered, and the cell fails to split. Otherwise, we use eq. 3 to calculate $T_{3}/T_{2}$. If this value is negative, the model predicts premature splitting; if it is positive, the model predicts successful division.

## SUPPLEMENTAL MATERIAL

Supplemental material mbio.01728-25-s0001.pdf Supplemental text, figures, and tables.

ASM does not own the copyrights to Supplemental Material that may be linked to, or accessed through, an article. The authors have granted ASM a non-exclusive, world-wide license to publish the Supplemental Material files. Please contact the corresponding author directly for reuse.
