# Supplementary material for: Cell wall mechanical stress could coordinate septal synthesis and scission in Staphylococcus aureus
Source: mBio. 2025 Oct 13;16(11):e01728-25. doi: 10.1128/mbio.01728-25 (PMC12607732; doi:10.1128/mbio.01728-25)
Supplement: Supplemental material — Supplemental text, figures, and tables. [file mbio.01728-25-s0001.pdf]

Supplementary Information: Cell wall mechanical stress could coordinate septal synthesis and scission in *Staphylococcus aureus*

Sheila Hoshyaripour,[1,2] Marco Mauri,[1,2] Jamie K. Hobbs,[3,4] Simon J. Foster,[5,4] and Rosalind J. Allen[1,2]

[1] *Theoretical Microbial Ecology, Institute of Microbiology, Faculty of Biological Sciences, Friedrich Schiller University Jena, Buchaer Strasse 6, 07745 Jena, Germany*

[2] *Cluster of Excellence Balance of the Microverse, Friedrich Schiller University Jena, Jena, Germany*

[3] *Department of Physics and Astronomy, University of Sheffield, Sheffield S37RH, United Kingdom*

[4] *The Florey Institute for Host-Pathogen Interactions, University of Sheffield, Sheffield S102TN, United Kingdom*

[5] *School of Biosciences, University of Sheffield, Sheffield S10 2TN, United Kingdom*

I. CALCULATION OF CIRCUMFERENTIAL STRESS IN PHASE 2 OF THE CELL CYCLE

Here we derive the circumferential stress distribution σ_h for a cell in phase 2 of the cell cycle, Eqs. 4 and 5 of the main text. The (half) cell is modelled as a thin hemispherical shell connected to a disc with an aperture (Figure S1B), representing the incomplete septum (Figure 1C of the main text). To calculate the stress distribution we use the approximate edge bending solution for spherical thin shells. In what follows we first provide an introduction to this solution method, then we show how it can be applied to various example cases relevant to our model, and finally we apply it to obtain the stress distribution for a cell in phase 2 of the cell cycle.

Approximate edge bending solution for spherical thin shells

If a thin shell is subjected to uniform internal pressure, as is the case in our model for phase 1 of the cell cycle, the shell expands uniformly and the tension in the shell is uniform. At equilibrium this leads to $P\pi a^2 = 2\pi a t \sigma$ (as shown in Figure S1A) and subsequently our result for phase 1, $\sigma = \frac{Pa}{2t}$.

However, if the shell cannot expand freely (e.g. due to geometrical constraints such as, in

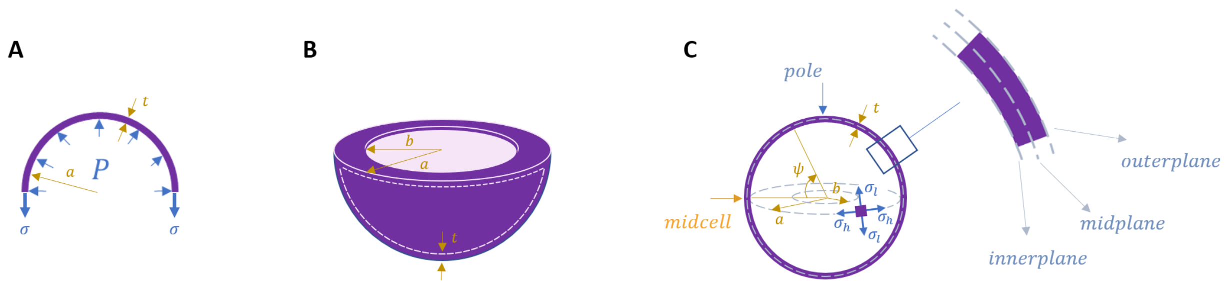

Figure S1. A) In phase 1 , stress in the cell wall (σ) balances the pressure, leading to the equation $P\pi a^2 = 2\pi a t \sigma$ B) In phase 2, the (half) cell is modelled as a hemispherical shell connected to a disc with an aperture b C) In phase 2 and 3, because of the presence of the septum, longitudinal (σ_l) and circumferential stress (σ_h) are not equal. The stresses also vary through the thickness and in this study we focus on the midplane. ψ shows the angle from the equator to the point on the shell where the stress is calculated, used in Eq. 4 in the main text (for the details of calculations see below)

pressure vessels, at the boundary between the cylinder and ends of the vessel, or in our model at the boundary between the peripheral wall and the septum), the shell has to bend locally to maintain its physical continuity. Here we explain how edge bending can be analyzed for a spherical shell. We use as our variable the angle φ from the vertical axis; lines of constant φ correspond to parallel circles on the shell and the edge of the shell corresponds to $\varphi = \alpha$ (Figure S2 A).

We start with the assumption that the thickness of the shell is small compared to its radius. This greatly simplifies our analysis since, for thin shells, approximate methods are available that give solutions with good accuracy. In the thin shell approximation, the equations of equilibrium given in detail in [1] can be reduced to:

$$\frac{d^2Q}{d\varphi^2} + \cot\varphi \frac{dQ}{d\varphi} - (\cot^2\varphi - \nu)Q = EtV\,, \tag{S1}$$

$$\frac{d^2V}{d\varphi^2} + \cot\varphi \frac{dV}{d\varphi} - (\cot^2\varphi + \nu)V = -\frac{a^2Q}{D}\,, \tag{S2}$$

where Q is the shearing force per unit length and V is the rotation of a tangent to a meridian (which is due to the shell element deformation (See [2] for more details)), while as in the main text, E, t and a denote the Young's modulus, shell thickness, and radius, respectively (we note V in these equations should not be confused with V_{cell} used for the volume of the cell). In Eq. S2, D is the flexural rigidity of the shell, which is given by

$$D = \frac{Et^3}{12(1-\nu^2)}\,. \tag{S3}$$

It can be shown [1] that, if the angle φ is large, the terms containing Q and V and their first derivatives can be neglected on the left-hand side of Eqs. S1 and S2, so that they simplify to:

$$\frac{d^2Q}{d\varphi^2} = EtV\,, \tag{S4}$$

and

$$\frac{d^2V}{d\varphi^2} = -\frac{a^2Q}{D}\,. \tag{S5}$$

V can be eliminated from these equations by substituting Eq. S4 into Eq. S5, resulting in

$$\frac{d^4Q}{d\varphi^4} + 4\lambda^4 Q = 0\,. \tag{S6}$$

where

$$\lambda^4 = 3(1-\nu^2)\left(\frac{a}{t}\right)^2\,. \tag{S7}$$

The general solution of this equation is

$$Q = C_1 e^{\lambda\varphi}\cos\lambda\varphi + C_2 e^{\lambda\varphi}\sin\lambda\varphi + C_3 e^{-\lambda\varphi}\cos\lambda\varphi + C_4 e^{-\lambda\varphi}\sin\lambda\varphi\,.$$

since we expect the stress to decrease as the angle φ decreases, only the first two terms need to be considered:

$$Q = C_1 e^{\lambda\varphi}\cos\lambda\varphi + C_2 e^{\lambda\varphi}\sin\lambda\varphi\,. \tag{S8}$$

C_1 and C_2 can be determined from the boundary conditions at the edge (where $\varphi = \alpha$). By introducing the angle $\psi = \alpha - \varphi$ (Figure S2A), the solution can be written as

$$Q = Ce^{-\lambda\psi}\sin(\lambda\psi + \gamma)\,. \tag{S9}$$

From Eq. S4, we can find V:

$$V = \frac{1}{Et}\frac{d^2Q}{d\varphi^2} = -\frac{2\lambda^2}{Et}Ce^{-\lambda\psi}\cos(\lambda\psi + \gamma)\,, \tag{S10}$$

and from V we can in turn obtain the bending moment M on the shell elements as a function of ψ [1]:

$$M = -\frac{D}{a}\frac{dV}{d\varphi} = \frac{a}{\lambda\sqrt{2}}Ce^{-\lambda\psi}\sin(\lambda\psi + \gamma + \frac{\pi}{4})\,. \tag{S11}$$

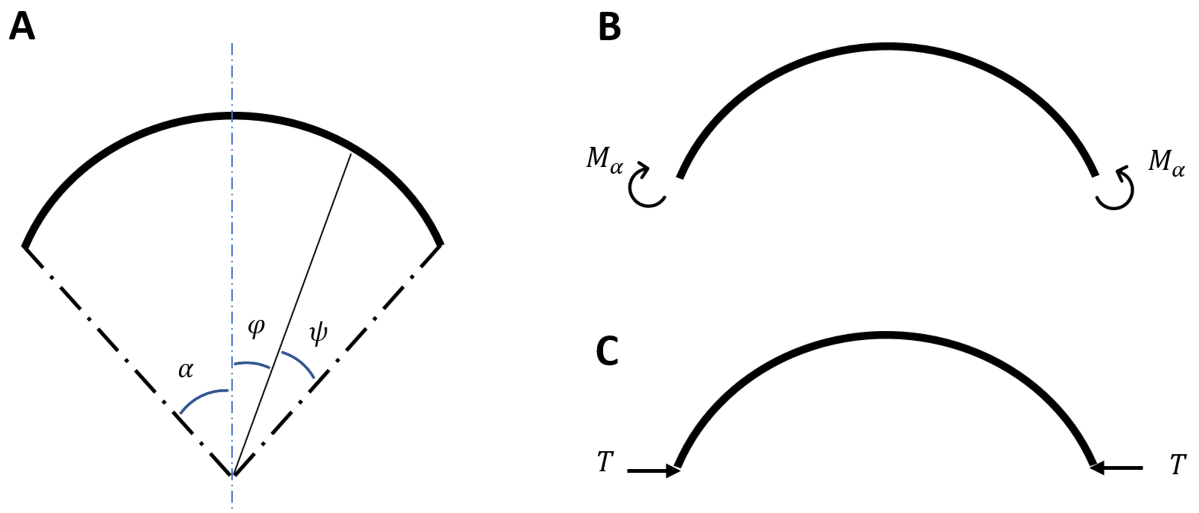

Figure S2. A) The angles used in the calculation, B, C) A bending moment and a force at the edge of a shell

The normal force N is then [1]:

$$N = -Q \cot \varphi = -\cot(\alpha - \psi) C e^{-\lambda \psi} \sin(\lambda \psi + \gamma) \,. \tag{S12}$$

The radial displacement δ of the shell (i.e. the deformation in the radial direction) as shown in [1] can be calculated as:

$$\delta \approx -\frac{a}{Et} \lambda \sqrt{2} C e^{-\lambda \psi} \sin(\lambda \psi + \gamma - \frac{\pi}{4}) \,. \tag{S13}$$

Specific examples: distributed bending moment or a distributed force

We now apply the analytical results for M, N, V and δ for a thin shell to two specific cases in which we have either a distributed bending moment or a distributed force. First, let us consider a shell with a distributed bending moment M_α, as shown in Figure S2b. In this case the boundary conditions are

$$(M)_{\varphi=\alpha} = M_\alpha \,, \tag{S14}$$

$$(N)_{\varphi=\alpha} = 0 \,. \tag{S15}$$

By substituting $\psi = 0$ into Eq. S12, we conclude that the boundary condition can be satisfied by taking $\gamma = 0$. Then, from Eq. S11, we must have:

$$C = \frac{M_\alpha 2\lambda}{a} \,. \tag{S16}$$

Inserting the values for γ and C into Eqs S2 and S13 we obtain

$$(V)_{\psi=0} = -\frac{4\lambda^3 M_\alpha}{Eat}\,, \tag{S17}$$

$$(\delta)_{\psi=0} = \frac{2\lambda^2 \sin\alpha}{Et} M_\alpha\,. \tag{S18}$$

Next, we consider a shell with a distributed tangential force T at the edges, as in Figure S2c. In this case, the boundary conditions are

$$(M)_{\varphi=\alpha} = 0\,, \tag{S19}$$

$$(N)_{\varphi=\alpha} = -T\cos\alpha\,. \tag{S20}$$

To satisfy the first condition we require $\gamma = -\frac{\pi}{4}$ and the second boundary condition leads to

$$C = -\frac{2T\sin\alpha}{\sqrt{2}}\,. \tag{S21}$$

Inserting these values of γ and C into Eqs. S2 and S13 leads to the results

$$(V)_{\psi=0} = \frac{2\lambda^2 \sin\alpha}{Et} T\,, \tag{S22}$$

$$(\delta)_{\psi=0} = -\frac{2\alpha\lambda\sin^2\alpha}{Et} T\,. \tag{S23}$$

Hemispherical shell with distributed bending moment and tangential force

Next we consider a hemispherical shell ($\alpha = \frac{\pi}{2}$) that has both a bending moment and tangential force at the edge. This problem can be solved by superposing the results derived above, leading to:

$$\begin{aligned} V &= -\frac{2\lambda^2}{Et}\frac{M_\alpha 2\lambda}{a} e^{-\lambda\psi}\cos(\lambda\psi) - \frac{2\lambda^2}{Et}\left(-\frac{2T}{\sqrt{2}}\right) e^{-\lambda\psi}\cos(\lambda\psi - \frac{\pi}{4}) \\ &= -\frac{4\lambda^3 M_\alpha}{Eat} e^{-\lambda\psi}\cos(\lambda\psi) + \frac{2\lambda^2 T}{Et} e^{-\lambda\psi}(\cos\lambda\psi + \sin\lambda\psi)\,, \end{aligned} \tag{S24}$$

$$\begin{aligned} M &= \frac{a}{\lambda\sqrt{2}}\frac{M_\alpha 2\lambda}{a} e^{-\lambda\psi}\sin\left(\lambda\psi + \frac{\pi}{4}\right) + \frac{a}{\lambda\sqrt{2}}\left(-\frac{2H}{\sqrt{2}}\right) e^{-\lambda\psi}\sin(\lambda\psi) \\ &= M_\alpha e^{-\lambda\psi}(\sin\lambda\psi + \cos\lambda\psi) - \frac{aH}{\lambda} e^{-\lambda\psi}\sin\lambda\psi\,, \end{aligned} \tag{S25}$$

$$\begin{aligned} \delta &= -\sqrt{2}\lambda\left(\frac{a}{Et}\right)\left(\frac{M_\alpha 2\lambda}{a}\right) e^{-\lambda\psi}\sin\left(\lambda\psi - \frac{\pi}{4}\right) - \sqrt{2}\left(\frac{a\lambda}{Et}\right)\left(-\frac{2T}{\sqrt{2}}\right) e^{-\lambda\psi}\sin\left(\lambda\psi - \frac{\pi}{2}\right) \\ &= \left(\frac{2\lambda^2 M_\alpha}{Et}\right) e^{-\lambda\psi}(\cos\lambda\psi - \sin\lambda\psi) - \left(\frac{2a\lambda T}{Et}\right) e^{-\lambda\psi}\cos\lambda\psi\,. \end{aligned} \tag{S26}$$

Applying the approximate edge bending solution to a cell in phase 2

We now show how these solutions can be used to calculate the stress distribution in the cell wall during phase 2 of the cell cycle. In phase 2, we consider half of the cell to be a hemisphere connected to a disc with an aperture representing the incomplete septum. Turgor pressure inside the cell causes both the septum and the peripheral cell wall to deform. In particular, the cell wall experiences a radial deformation which produces a force T (Figure 1C in the main text and Figure S2C) at the boundary between the peripheral wall and the septum, leading to radial deformation also in the septum. Correspondingly, a reaction force (equal but in the opposite direction) is exerted by the stretched septum on the peripheral wall.

SEM images of newly split *S. aureus* cells suggest that the septum is initially flat (although it later bends, eventually producing a rounded daughter cell) [3]. Therefore in our model we assume that the septum is flat. This implies that the rotation angle V of the cell wall is zero at the edge. Therefore a bending moment (M_α) must act on the peripheral wall to compensate for the force T, and the bending force must have an equal and opposite reaction that acts on the septum (as shown in Figure 1C of the main text).

To calculate the stress in the cell wall we superpose the effects of pressure, force and bending moment. At the edge (i.e. at $\psi = 0$ in our hemispherical shell) the rotation angle is zero as discussed above. Therefore Eq. S24 gives:

$$(V)_{\psi=0} = -\frac{4\lambda^3}{E_w a t} M_\alpha + \frac{2\lambda^2}{E_w t} T = 0\,. \tag{S27}$$

It follows that

$$M_\alpha = \frac{a}{2\lambda} T\,.$$

Inserting this result into Eqs. S25 and S26, the bending moment M and deformation δ can be written as

$$\begin{aligned} M &= \frac{aT}{2\lambda} e^{-\lambda\psi}(\sin\lambda\psi + \cos\lambda\psi) - \frac{aT}{\lambda} e^{-\lambda\psi}\sin\lambda\psi \\ &= \frac{aT}{2\lambda} e^{-\lambda\psi}(\cos\lambda\psi - \sin\lambda\psi)\,, \end{aligned} \tag{S28}$$

$$\begin{aligned} \delta &= \frac{2\lambda^2}{E_w t}\frac{aT}{2\lambda} e^{-\lambda\psi}(\cos\lambda\psi - \sin\lambda\psi) - \frac{2a\lambda T}{E_w t} e^{-\lambda\psi}\cos\lambda\psi \\ &= -\frac{a\lambda T}{E_w t} e^{-\lambda\psi}(\sin\lambda\psi + \cos\lambda\psi)\,. \end{aligned} \tag{S29}$$

In the longitudinal direction, the stress is calculated by summing the pressure contribution and the effect of the bending moment. The stress in the inner and outer planes of the hemispherical peripheral wall is [4]:

$$\sigma_l = \frac{Pa}{2t} \pm \frac{6}{t^2}M\,, \tag{S30}$$

where the positive sign corresponds to the inner plane (the inside of the cell wall) and the negative sign corresponds to the outer plane (the outside of the cell wall) (Figure S1C). In the circumferential (hoop) direction, the stress encompasses contributions from the pressure, the radial deformation δ that is different at each location and the lateral effect of the bending moment due to Poisson's effect [4]:

$$\sigma_h = \frac{Pa}{2t} + E_w\frac{\delta}{a} \pm \nu\frac{6}{t^2}M\,. \tag{S31}$$

Eqs. S30 and S31 give the stress in the longitudinal and circumferential directions at any angle ψ on the cell wall (see Figure S2A), in the outer and inner planes. In the midplane of the cell wall, we only observe the effect of the pressure and (for circumferential stress) the effect of radial constriction:

$$\sigma_l = \frac{Pa}{2t}\,, \tag{S32}$$

$$\sigma_h = \frac{Pa}{2t} - \frac{\lambda T}{t}e^{-\lambda\psi}(\sin\lambda\psi + \cos\lambda\psi)\,, \tag{S33}$$

where we have used Eq. S29. Therefore the presence of the septum does not alter the longitudinal stress in the midplane of the cell wall but it does alter the stress distribution in the circumferential direction. For this reason we focus on the circumferential (hoop) stress in this study.

To calculate the circumferential stress from Eq. S33, we need to know the edge force T. Continuity of the material at the edge implies that

$$\delta_{wp} + \delta_{wf} = \delta_s\,, \tag{S34}$$

where δ_{wp} and δ_{wf} are the radial deformation of the cell wall at the edge due to the pressure and due to the edge forces, respectively, and δ_s is the radial deformation of the septum. δ_{wp} can be calculated using Hooke's law:

$$\begin{aligned}\delta_{wp} &= a\frac{1}{E_w}(\sigma_2 - \nu\sigma_1)\,,\\ &= a\frac{1}{E_w}(\frac{Pa}{2t} - \nu\frac{Pa}{2t})\,,\\ &= \frac{Pa^2(1-\nu)}{2E_wt}\,.\end{aligned} \tag{S35}$$

Eq. S29 can be used to find δ_{wf}:

$$\delta_{wf} = -\frac{a\lambda T}{E_w t}\,. \tag{S36}$$

To obtain δ_s, the deformation of the incomplete septum, we consider a thin disc with a hole, where the stress at the inner boundary is equal to the pressure ($\sigma_{r_i} = \sigma_r(b) = -P$) and the stress at the outer boundary is T divided by the thickness of the septum ($\sigma_{r_o} = \sigma_r(a) = \frac{T}{t_s}$). The deformation of this disk at the outer edge is (see Section III below):

$$\delta_s = (u)_{r=a} = \left(\frac{1-\nu}{E_s}\right)\left(\frac{\frac{T}{t_s}a^2 + Pb^2}{a^2 - b^2}\right)a + \left(\frac{1+\nu}{E_s}\right)\left(\frac{(\frac{T}{t_s} + P)a^2b^2}{a^2 - b^2}\right)\left(\frac{1}{a}\right)\,. \tag{S37}$$

Substituting Eqs. S35, S36 and S37 into Eq. S34 we can write

$$\frac{Pa^2(1-\nu)}{2E_w t} - \frac{a\lambda}{E_w t}T = \left(\frac{1-\nu}{E_s}\right)\left(\frac{\frac{T}{t_s}a^2 + Pb^2}{a^2 - b^2}\right)a + \left(\frac{1+\nu}{E_s}\right)\left(\frac{(\frac{T}{t_s} + P)a^2b^2}{a^2 - b^2}\right)\left(\frac{1}{a}\right)\,, \tag{S38}$$

which gives the following expression for the force T:

$$T = \frac{\frac{Pa^2(1-\nu)}{2E_w t} - \frac{2Pab^2}{E_s(a^2-b^2)}}{\left(\frac{1-\nu}{E_s}\right)\left(\frac{a^3}{t_s(a^2-b^2)}\right) + \left(\frac{1+\nu}{E_s}\right)\left(\frac{ab^2}{t_s(a^2-b^2)}\right) + \frac{a\lambda}{E_w t}}\,. \tag{S39}$$

Assuming that the septum grows in both thickness and radius and that the thickness of the complete septum is equal to the thickness of the wall, i.e. $t_s = t(a-b)/a$ where b is the aperture size (see Section IV), and defining the relative septum stiffness $E_r = E_s/E_w$, we arrive at the expression for T given in Eq. 5 of the main text:

$$T = \frac{\frac{Pa^2(1-\nu)}{2t}E_r - \frac{2Pab^2}{(a^2-b^2)}}{(1-\nu)\frac{a^4}{t(a-b)(a^2-b^2)} + (1+\nu)\frac{a^2b^2}{t(a-b)(a^2-b^2)} + \frac{a\lambda E_r}{t}}\,. \tag{S40}$$

Now, using Eq. S33, the circumferential stress can be written as

$$\sigma_h = \frac{Pa}{2t} - \frac{\lambda}{t}\left(\frac{\frac{Pa^2(1-\nu)}{2t}E_r - \frac{2Pab^2}{(a^2-b^2)}}{(1-\nu)\frac{a^4}{t(a-b)(a^2-b^2)} + (1+\nu)\frac{a^2b^2}{t(a-b)(a^2-b^2)} + \frac{a\lambda E_r}{t}}\right) \times e^{-\lambda\psi}(\sin\lambda\psi + \cos\lambda\psi)\,. \tag{S41}$$

Eq. S41 is used to calculate the circumferential stress in the cell wall in Figure 2A of the main text. Since the aperture size b decreases to zero during phase 2 of the cell cycle, this equation predicts how the cell wall stress changes as septum synthesis progresses.

Circumferential stress in phase 3

The same approach is used to calculate the stress in phase 3. Here, since the septum has been completed, the aperture size b is zero. In this case, the deformation of the septum is

$$\delta_s = \frac{a(1-\nu)}{E_s t}T \tag{S42}$$

where the thickness of the complete septum is taken to be equal to the thickness t of the cell wall. The continuity equation S34 can be used to calculate T. Substituting Eqs. S35, S36 and S42 into Eq. S34, we can write:

$$\frac{Pa^2(1-\nu)}{2E_w t} - \frac{a\lambda}{E_w t}T = \frac{a(1-\nu)}{E_s t}T\,, \tag{S43}$$

which gives the force T:

$$T = \frac{Pa(1-\nu)}{\frac{2(1-\nu)}{E_r} + 2\lambda}\,.$$

The circumferential stress in phase 3 can then be written as

$$\sigma_h = \frac{Pa}{2t} - \frac{Pa}{2t}\left(\frac{\lambda E_r}{1+\frac{\lambda E_r}{(1-\nu)}}\right)e^{-\lambda\psi}(\sin\lambda\psi + \cos\lambda\psi)\,. \tag{S44}$$

Focusing on the stress at the cell equator ($\psi = 0$), and assuming $\nu \simeq 0.5$ as is typical for soft biological tissue, we obtain the following result for the equatorial hoop stress in phase 3:

$$\sigma_h(\psi = 0) = \frac{Pa}{2t}\left(\frac{1+\lambda E_r}{1+2\lambda E_r}\right)\,. \tag{S45}$$

An equivalent expression is derived in [5]. Since $(1+\lambda E_r)/(1+2\lambda E_r) < 1$, the presence of the septum reduces the circumferential stress in the peripheral wall at the equator, where the wall and septum meet.

Stress distribution through the cell wall

In this study we focus on the stress in the midplane of the wall. However, due to the bending moment, the stress is not uniform through the thickness of the cell wall. The longitudinal and circumferential stresses in the inner and outer planes of the cell wall can be calculated using Eqs. S30 and S31. Figure S3 shows the longitudinal and circumferential stress distributions in the inner, outer and midplanes of the cell wall in phase 3 of the cell cycle, for a wild-type (MSSA) cell as a function of the angle ψ (for parameters see Table 1 of the main text). The stress in phase 3 is compared to that in phase 1. Around the division site ($\psi = 0$), the stress is lower in the outer plane and higher in the inner plane.

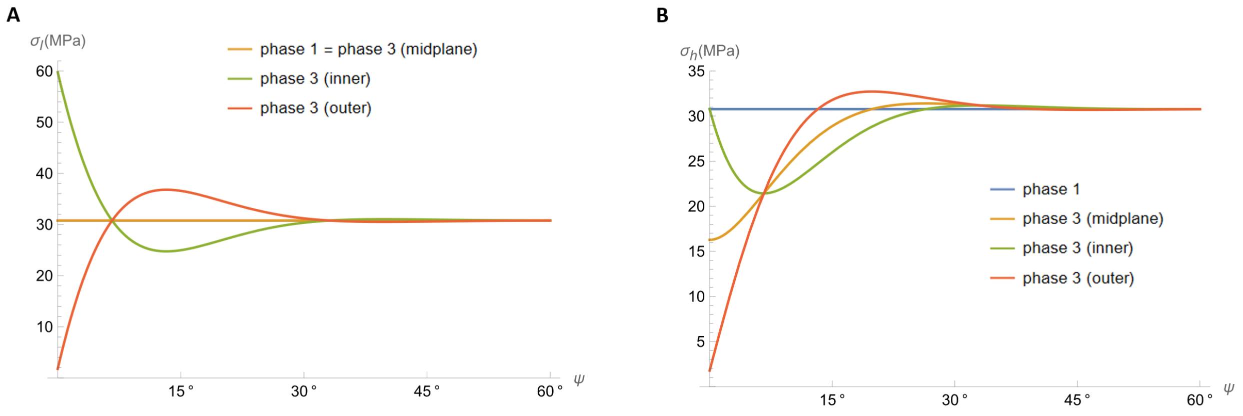

Figure S3. Stress distribution in the inner, outer and midplane calculated for a wild-type (MSSA) cell (for parameters see Table 1 of the main text). The stress distribution is calculated in phase 3 of the cell cycle and compared to that in phase 1 (note that in phase 1 the stress is uniform through the wall). The upper plot (A) shows longitudinal stress; the lower plot (B) shows circumferential stress.

II. NOTES ON VARIABLES AND PARAMETERS

Cell radius in the pressurized and depressurized states

In our model, the variable a corresponds to the radius of a depressurized cell, i.e. a cell without turgor pressure. Turgor pressure inflates the cell, causing the radius to increase to a value a_P that is given by

$$a_P = a + \frac{Pa^2(1-\nu)}{2Et} \,. \tag{S46}$$

where E is the Young's modulus of the cell wall, treated as a homogeneous material and, as elsewhere, P is the turgor pressure, t is the cell wall thickness and ν is Poisson's ratio.

To obtain parameter values for the radius a in our model we use literature data on the volume of live cells, V_{cell}. These measurements are of course made in the presence of turgor. Therefore from the measured volume V_{cell} we obtain the pressurized cell radius a_P by

$$a_P = \sqrt[3]{\frac{3V_{cell}}{4\pi}} \,. \tag{S47}$$

We then use Eq. S46 to obtain the values of the depressurized radius a listed in Table 1 of the main text, as input to the model. Also the aperture size b corresponds to the unpressurized case. To facilitate connection with biological reality, in Figures 5, 6, 7 and 8 of the main text, the horizontal axis shows the "live cell radius", i.e. the pressurized radius a_P.

Young's modulus of the cell wall

In order to use Eq. S46 to calculate the depressurized cell radius we need a value for the Young's modulus E of the cell wall material (which is here assumed to be homogeneous). In a previous model [5], E was estimated to be two orders of magnitude greater than the turgor pressure. Taking the turgor pressure to be around 2 MPa [6], this would suggest that the Young's modulus of the cell wall is on the order of hundreds of MPa. Reported experimental values for E vary widely [7, 8] and seem to depend strongly on the measurement method and probably also on the bacterial strain and growth conditions. However, values in the range of hundreds of MPa were obtained for measurements of the mechanical properties of peptidoglycan (PG) in a cell-separation deficient mutant of *Bacillus subtilis* [9]. This mutant forms a bacterial "thread" consisting of densely packed and aligned filamentous cells; the mechanical response of the bacterial thread was used to infer the mechanical properties of PG at different humidities. Here we use the reported value of 200 MPa that was obtained at 65 percent humidity [9].

It is important to note that, in our calculations, the value of E affects only the conversion between pressurized and depressurized cell radius. All other calculations (e.g. the stress distribution and the aperture size at the time the autolysins are triggered) require only the parameter E_r, i.e. the stiffness of the septum relative to the cell wall. E_r is assumed to be 1.2, as explained in the main text.

We also note that for the calculations with methicillin (Figure 8 of the main text), we neglected any changes in E or E_r that might be caused by methicillin.

III. CALCULATION OF STRESS IN THE SEPTUM

Our study focuses on the stress distribution in the peripheral cell wall (especially close to the division site), but we can also calculate the stress distribution in the septum. For a thin disk of outer radius a, containing a hole with inner radius b, the stresses in the radial and tangential directions (which we denote σ_r and σ_t) can be written as [10]:

$$\sigma_r = C_1 - \frac{C_2}{r^2}\,, \tag{S48}$$

$$\sigma_t = C_1 + \frac{C_2}{r^2}\,, \tag{S49}$$

where r is the radial coordinate and C_1 and C_2 are constants to be determined from the boundary conditions.

In our problem, the radial stress at the inner boundary $r = b$ is equal to the turgor pressure ($\sigma_{r_i} = \sigma_r(b) = -P$) and the radial stress at the outer boundary is given by T divided by the thickness of the septum ($\sigma_{r_o} = \sigma_r(a) = \frac{T}{t_s}$). With these two boundary conditions, we can determine the values of C_1 and C_2:

$$C_1 = \frac{Pb^2 + (T/t_s)a^2}{a^2 - b^2}\,, \tag{S50}$$

$$C_2 = \frac{a^2b^2(P + T/t_s)}{a^2 - b^2}\,. \tag{S51}$$

The radial deformation u of the septum can be obtained as a function of r [10]:

$$u = \frac{C_1(1-\nu)}{E_s} r + \frac{C_2(1+\nu)}{E_s}\left(\frac{1}{r}\right) \tag{S52}$$

which, on inserting the solutions for C_1 and C_2, gives:

$$u = \left(\frac{1-\nu}{E_s}\right)\left(\frac{Pb^2 + \frac{T}{t_s}a^2}{a^2 - b^2}\right) r + \left(\frac{1+\nu}{E_s}\right)\left(\frac{a^2b^2(P + \frac{T}{t_s})}{a^2 - b^2}\right)\left(\frac{1}{r}\right)\,. \tag{S53}$$

Figure S4 shows the predicted patterns of radial and tangential stress on the partially and fully completed septum. During phase 2, the tangential stress, which is always positive, increases as we move inwards, being maximal at the inner boundary. In contrast, the radial stress is negative at the inner boundary because of compression caused by the turgor pressure; however as we move outwards it becomes positive, indicating tension, because of the outward forces exerted by the peripheral wall on the septum. There is therefore an intermediate radius where the radial stress is zero. During phase 3 both the radial and tangential stresses are constant across the septum.

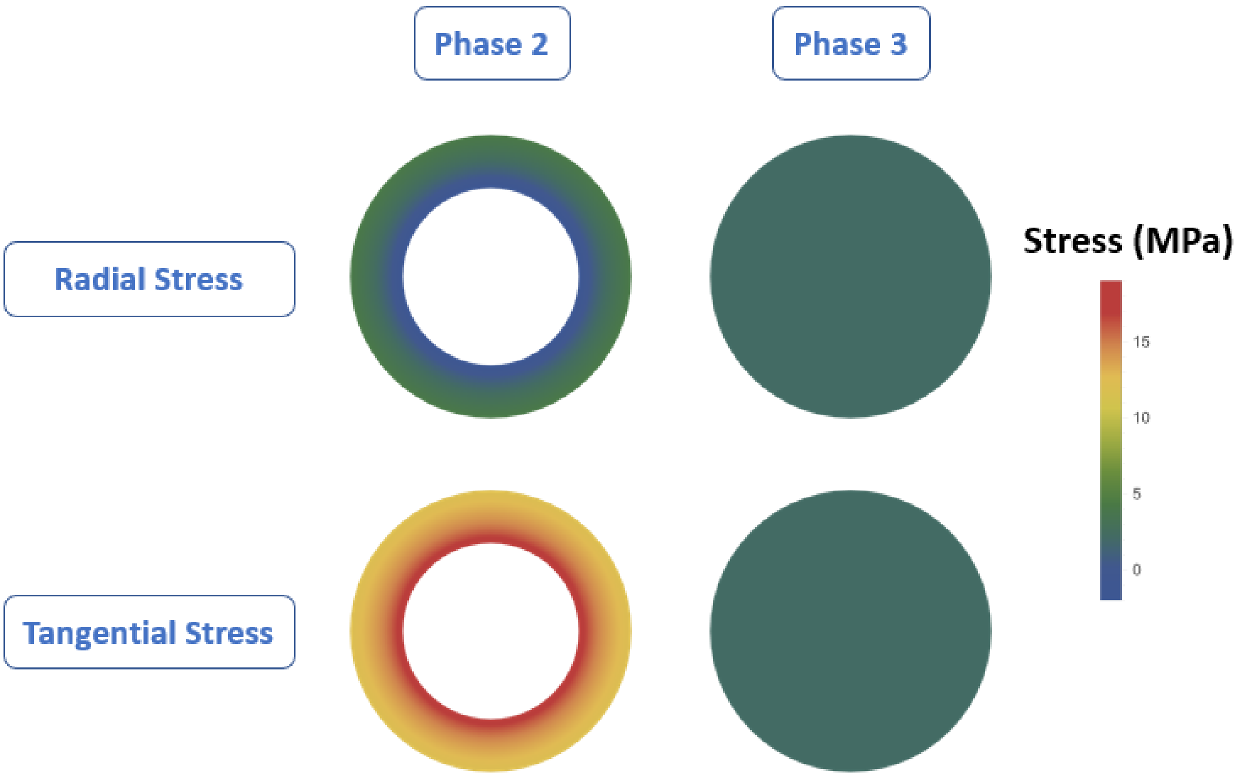

Figure S4. Distribution of radial and tangential stresses in the incomplete septum (phase 2; shown for the case where $(a-b)/a = 3/8$) and the complete septum (phase 3).

IV. MODELLING SEPTAL GROWTH

In this work, we have assumed that the septum increases in thickness as it grows in the radial direction (Figure S5). More specifically, we assume that the septal thickness t_s is given by

$$t_s = t(a-b)/a\,, \tag{S54}$$

where, as before, a is the unpressurized cell radius, b is the aperture size (which also refers to an unpressurized cell) and t is the thickness of the completed septum (when $b = 0$), which is equal to the thickness of the peripheral cell wall. Eq. S54 corresponds to a picture in which the thickness of the septum is uniform, but increases during the cell cycle as the aperture size decreases, as illustrated in Figure S5.

In reality, it is likely that the incomplete septum is more wedge-shaped, being thicker on the outside, close to the peripheral wall, but thinner at its inner edge [11]. To account for this, we can extend our calculation to the general case of a disk of radius a with an aperture of radius b,

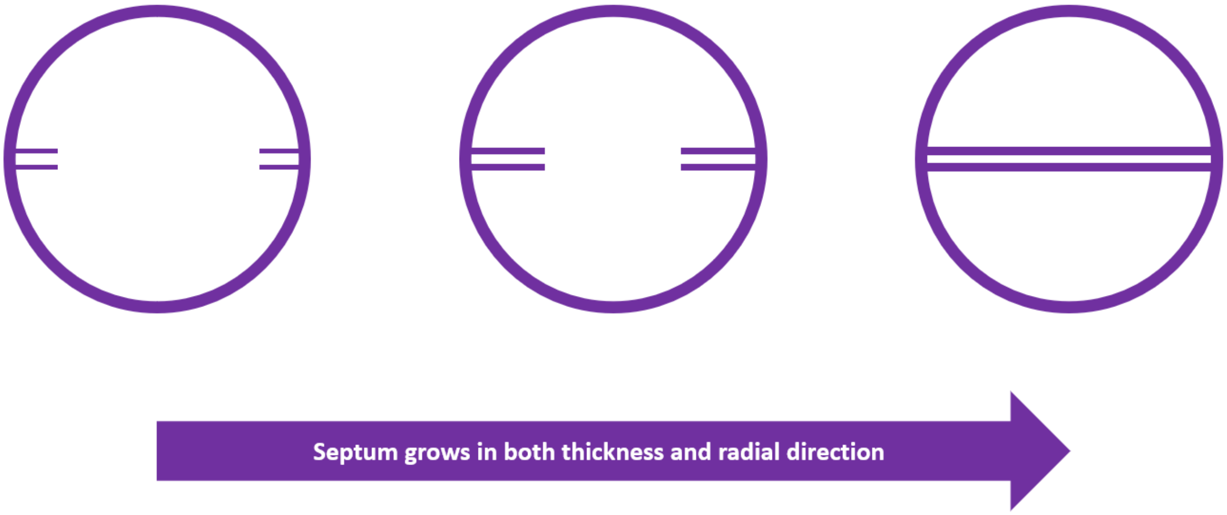

Figure S5. Illustration of our model for septal growth during phase two. We assume that the septum increases in thickness during phase 2, proportional to its growth in the radial direction. However an alternative model in which the septum grows as a wedge gives similar results (see text).

in which the thickness h of the disk is a linearly increasing function of the radial coordinate r: $h(r) = t(a-b)r/a^2$, where t is the thickness of the disk at its outer edge ($r = a$). Assuming that the stresses do not vary over the thickness of the disk, the equation describing force equilibrium for an element at radial position r in the disk is [2]:

$$\frac{d}{dr}(hr\sigma_r) - h\sigma_\theta = 0\,. \tag{S55}$$

This equation is satisfied by defining a function $F(r)$ such that $hr\sigma_r = F(r)$ and $h\sigma_\theta = \frac{dF}{dr}$. Hooke's law with a plane stress assumption implies that the radial and tangential strains are given by $\epsilon_r = \frac{1}{E_s}(\sigma_r - \nu\sigma_\theta)$ and $\epsilon_\theta = \frac{1}{E_s}(\sigma_\theta - \nu\sigma_r)$. By replacing $\sigma_r = \frac{F(r)}{hr}$ and $\sigma_\theta = \frac{dF}{hdr}$ and inserting them into the the compatibility equation, $\epsilon_\theta - \epsilon_r - r\frac{d\epsilon_\theta}{dr} = 0$, we obtain:

$$r^2\frac{d^2F}{dr^2} + r\frac{dF}{dr} - F - \frac{rdh}{hdr}(r\frac{dF}{dr} - \nu F) = 0\,. \tag{S56}$$

By replacing $h(r) = t(a-b)r/a^2$, we have:

$$r^2\frac{d^2F}{dr^2} - (1-\nu)F = 0\,. \tag{S57}$$

Assuming $\nu \simeq 0.5$ and solving the differential equation, we obtain:

$$F(r) = Ar^\alpha + Br^\beta \tag{S58}$$

where $\alpha, \beta = \frac{1 \pm \sqrt{3}}{2}$. Eq. S58 allows the stresses in the septum, σ_r and σ_θ, and consequently also the strains ϵ_r and ϵ_θ to be obtained.

We can then calculate the stress in the peripheral cell wall by the same approach outlined in Section I above, but replacing Eq. S37 for the radial deformation at the outer edge of the disk by the radial deformation of this alternative septum model ($r\epsilon_\theta$ at $r = a$).

Figure S6 compares the predicted circumferential stress in the peripheral cell wall at the division site, for the two septal growth models outlined here - i.e. for the model with uniform septal thickness that increases as b decreases, and the model with a wedge-shaped septum. The predictions of the two models are very similar, although the value of the aperture size at which autolysins are predicted to the triggered differs slightly between the models; autlolysins are triggered somewhat later for the wedge-shaped septum model.

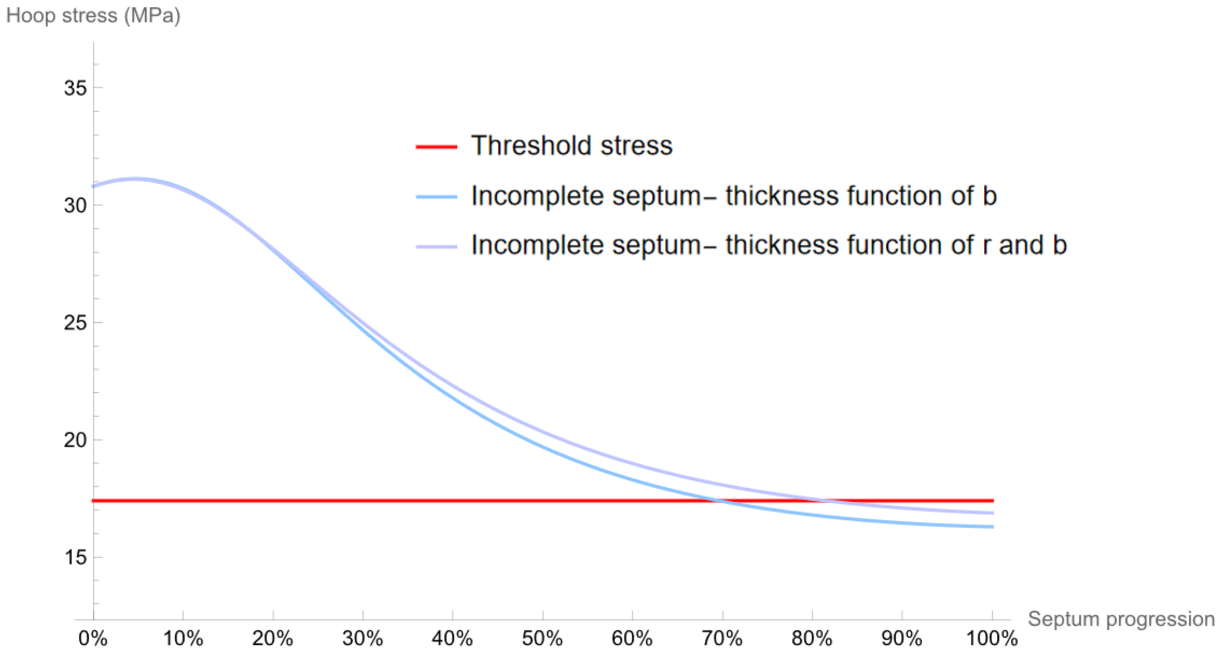

Figure S6. The effect of septum growth model on the predicted circumferential cell wall stress at the division site, as the septum is formed. Results are plotted for the parameter set corresponding to MSSA (see Table 1 of the main text). The red line indicates the threshold stress value for triggering of autolysin activity, as described in the main text.

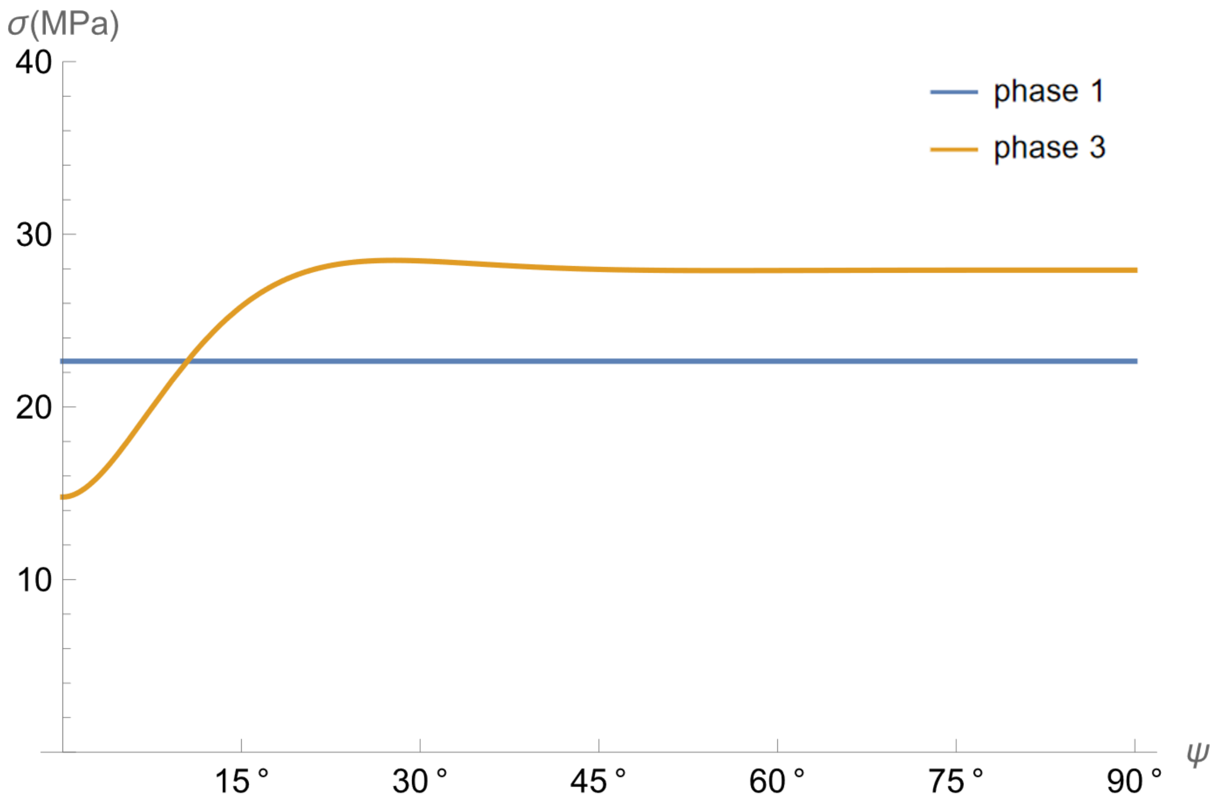

Figure S7. Circumferential stress in MSSA cell wall as a function of angle ψ, for a MSSA cell in phase 1 and 3 with the cell size reported in [3]. Other parameters as for MSSA (see Table 1 of the main text).

V. MODELLING A GROWING SPHERICAL CELL

In this study, we have assumed that the cell size remains constant during the cell cycle. To show that our model is still valid for a growing sphere, we compare the stresses in MSSA in phase 1 and phase 3 using the cell size reported in [3]. Figure S7 shows that, even in the presence of growth, stress around the division site in phase 3 is lower than the stress in phase 1. However, it is worth mentioning that even if stresses around the division site in phase 3 were not lower than stresses in phase 1, this was not against our model, since autolysins also need to be localized around the division site to be able to get activated by lower stress and they might not be localised before getting close to the end of the cycle.

VI. MODELLING A GROWING ELLIPSOIDAL CELL

In this study, we have modelled the *S. aureus* cell as a pressurized sphere; this implies that the stress in the cell wall is uniform during phase 1 of the cell cycle. However, in reality *S. aureus* cells are somewhat ellipsoidal [3], implying a non-uniform stress pattern in the peripheral wall, even in phase 1. Up to now we have also neglected to account for changes in cell size during the cell cycle. In reality, the cell elongates, becoming more ellipsoidal as the cell cycle progresses [3]. At the beginning of Phase 1, it has been reported that the cell has semi-major and semi-minor axes of 0.52 ± 0.03 μm and 0.46 ± 0.03 μm, respectively [3]. Just before splitting, at the end of Phase 3, the semi-major and semi-minor axes are 0.70 ± 0.04 μm and 0.55 ± 0.03 μm respectively. In a pressurized ellipsoidal shell the stress is higher at the equator and lower at the poles, hence the circumferential stress around the division site in phase 2 is expected to be higher compared to that of a spherical cell, potentially cancelling the decrease in stress caused by septum formation. Therefore it is important to understand whether the mechanical trigger model still works for an ellipsoidal cell.

To this end, we now calculate the stresses in an ellipsoid cell in phase 1 and phase 3, accounting for the change in cell geometry due to growth [3]. We define a_1 as the radius in the direction of the pole and a_2 as the equatorial radius. The ellipse is described by the following equation:

$$a_1^2 x^2 + a_2^2 y^2 = a_1^2 a_2^2 \,. \tag{S59}$$

For a pressurized ellipsoidal thin shell the longitudinal and circumferential stresses are given by [4]

$$\sigma_l = \frac{P r_2}{2t} \,, \tag{S60}$$

$$\sigma_h = \frac{P}{t}\left(r_2 - \frac{r_2^2}{2r_1}\right) \,, \tag{S61}$$

where

$$r_1 = \frac{(a_2^4 y^2 + a_1^4 x^2)^{\frac{3}{2}}}{a_2^4 a_1^4} \,, \tag{S62}$$

$$r_2 = \frac{(a_2^4 y^2 + a_1^4 x^2)^{\frac{1}{2}}}{a_1^2} \,. \tag{S63}$$

At the pole, $r_1 = r_2 = a_2^2/a_1$, leading to the following result for the stresses:

$$\sigma_l(\text{pole}) = \sigma_h(\text{pole}) = \frac{P a_2^2}{2a_1 t} \,. \tag{S64}$$

At the equator, $r_1 = a_1^2/a_2$ and $r_2 = a_2$, leading to:

$$\sigma_l(\text{equator}) = \frac{Pa_2}{2t}\,, \tag{S65}$$

$$\sigma_h(\text{equator}) = \frac{Pa_2}{t}\left(1 - \frac{a_2^2}{2a_1^2}\right)\,. \tag{S66}$$

The deformation of the ellipsoid at the equator is given by

$$\delta_e = a_2\left(\frac{\sigma_h}{E} - \frac{\nu\sigma_l}{E}\right) = \frac{Pa_2^2}{Et}\left(1 - \frac{a_2^2}{2a_1^2} - \frac{\nu}{2}\right)\,. \tag{S67}$$

In phase 1, we calculate the circumferential stress at the pole and at the equator using Eqs. S64 and S66. To calculate the stresses in phase 3 of the cell cycle, we need to also take account of the presence of the septum. Therefore the equations derived above need to be combined with the approximate edge-bending solution [4], as explained in Section I for the spherical case.

Figure S8 shows the prediction for the cirumferential stress distribution in the cell wall (as a function of the angle ψ; see Figure S2A), for an elliptical MSSA cell in phases 1 and 3 of the cell cycle. As expected, in phase 1 the stress is higher at the cell equator compared to the pole. In phase 3, the stress pattern is somewhat different for the ellpisoidal cell compared to its spherical counterpart, showing a maximum at intermediate ψ rather than at the pole as for a spherical cell (see the images in Figure 2 of the main text). However the circumferential stress is still lower around the division site than anywhere else in the cell wall in phase 3, suggesting that the mechanical trigger model should still function.

It is interesting to speculate that modulating its elongation might provide a way for the cell to tune the stress around the division site and hence the timing of autolysin activation.

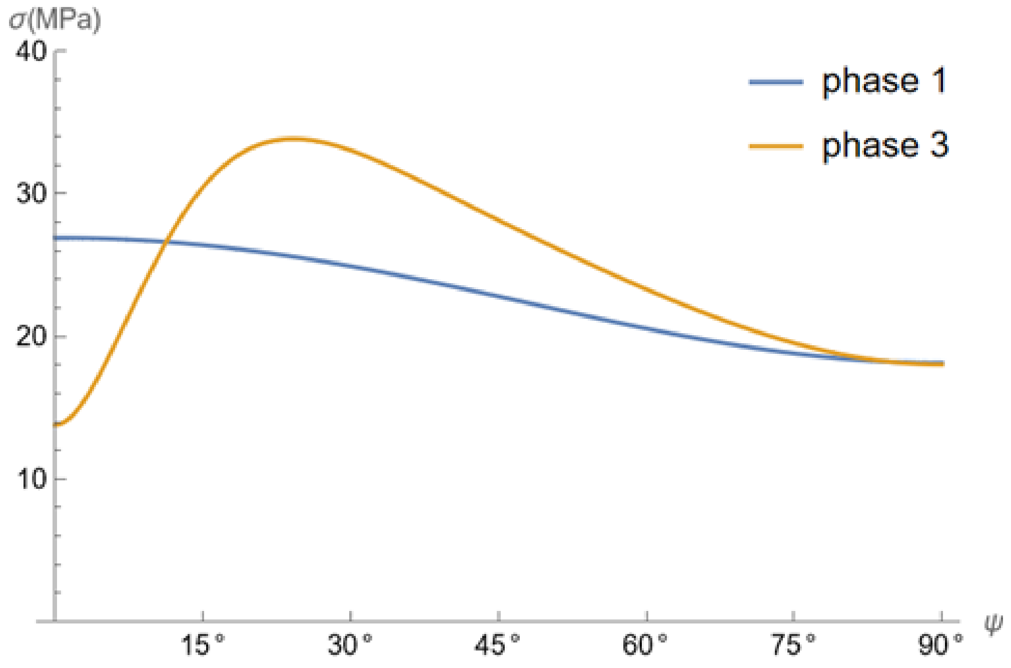

Figure S8. Circumferential stress in the cell wall as a function of angle ψ, for an elliposidal MSSA cell with the following dimensions: Phase 1 $a_1 = 0.52 \pm 0.03$ μm $a_2 = 0.46 \pm 0.03$ μm; phase 2 $a_1 = 0.70 \pm 0.04$ μm, $a_2 = 0.55 \pm 0.03$ μm [3]. Other parameters as for MSSA (see Table 1 of the main text).

VII. THE ROLE OF SEPTUM STIFFNESS

In this work, we have taken the stiffness of the septal material to be 1.2 times that of the wall (parameter E_r; see Table 1 of the main text). This motivated by two observations: firstly, atomic force microscopy measurements of the stiffness of newly exposed septal material versus the older cell wall [12] and secondly, observations that the newly synthesized septum consists of a dense PG mesh adorned with with tightly packed concentric rings, while the peripheral cell wall has a dense mesh on the inside transitioning to an open mesh structure towards the outside [13].

The relative stiffness of the septum and the peripheral wall may change, e.g. under antibiotic treatment. For example, the septal ring structure disappears in the presence of methicillin [14], which suggests a lower septum stiffness [12]. Therefore it is interesting to investigate how the cell fate predictions of the mechanical trigger model depend on the parameter E_r. Figure S9 shows a cell fate map for MSSA cells (see Table 1 of the main text), plotted as a function of the live cell radius a_P and the septal stiffness parameter E_r. Depending on the cell size, the fate of the cell may be more or less sensitive to the stiffness of the septum. However, our parameter sensitivity analysis (see Section VIII below) shows that the cell size and cell wall thickness have a greater impact on

the cell's ability to divide than the septal stiffness.

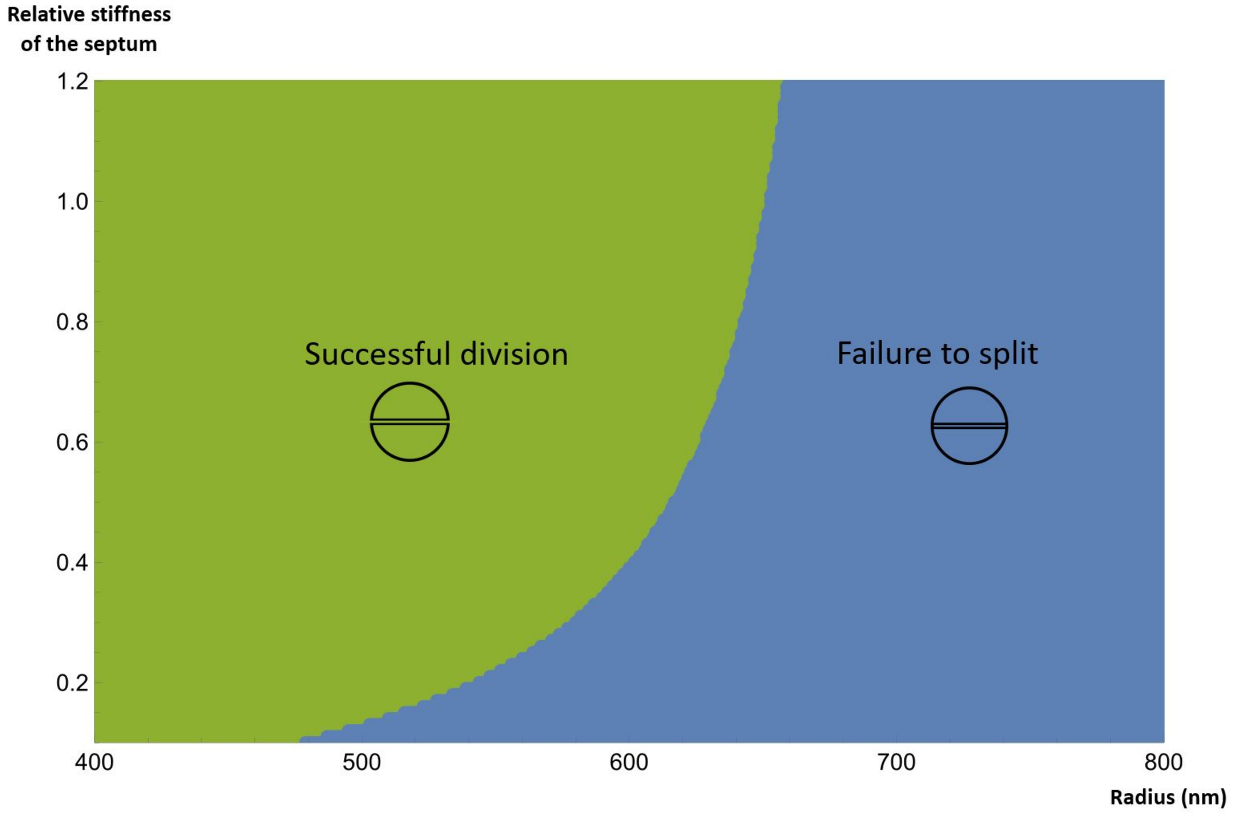

Figure S9. Cell fate map illustrating the role of septum stiffness. The map is plotted as a function of the live cell radius (a_P) and the stiffness of the septum relative to the peripheral wall (E_r). The other model parameters are those of MSSA cells (see Table 1 of the main text). The green region shows parameter values where the mechanical trigger model predicts successful division; in the blue region the model predicts failure to split.

VIII. PARAMETER SENSITIVITY ANALYSIS

	model outcome	c_p	c_a	c_t	c_{E_r}
MSSA	Stress in phase 1	1	1	-1	0
	Stress in phase 2	1	0.93	-0.93	-0.07
	Stress in phase 3	1	0.97	-0.97	-0.05
MRSA	Stress in phase 1	1	1	-1	0
	Stress in phase 2	1	0.89	-0.89	-0.11
	Stress in phase 3	1	0.97	-0.97	-0.06

Table S1. Parameter sensitivity analysis: sensitivity coefficients for the dependence of the equatorial stress in the circumferential direction, close to the division site, for MSSA and MRSA cells in phases 1, 2 and 3 of the cell cycle (assuming 50% septum completion in phase 2), on the parameters P, a, t and E_r.

To analyze the dependence of the calculated mechanical stress patterns to the parameters of our model, we performed a parameter sensitivity analysis. In general the outcome of a model can be expressed as $y = f(x)$, where y is the outcome of interest and x is a parameter of the model. The associated sensitivity coefficient $c_x = \frac{x}{y}\frac{\partial y}{\partial x}$ is a dimensionless number whose absolute value expresses the relative change of y associated with a variation in x. The closer c_x is to zero, the less sensitive the system is to the parameter x. Negative values show that the outcome y depends inversely on the parameter x, i.e. the outcome decreases as x increases.

Table S1 shows sensitivity coefficients for the predicted stress in the peripheral wall at the cell equator, close to the division site, for MSSA and MRSA cells in phases 1, 2 and 3 of the cell cycle (using as $f(x)$ Eqs 1 and 2 of the main text, with the parameter values in Table 1 of the main text and, for phase 2, assuming that the septum is half complete). The sensitivity coefficients are computed with respect to the turgor pressure P, the cell radius a, the cell wall thickness t and the septum relative stiffness E_r (i.e. these parameters play the role of x in the analysis). The analysis reveals a strong dependence of the calculated stress on the turgor pressure, cell radius and cell wall thickness, but only a much weaker dependence on the relative septal stiffness. As one would expect (e.g. from Eqs. 1-3 of the main text) the stress depends linearly on the turgor pressure, leading to a sensitivity coefficient $c_P = 1$. The dependence on cell radius is close to linear and the dependence

on cell wall thickness is also close to linear but negative.

---

[1] S. Timoshenko and S. Woinowsky-Krieger, *Theory of plates and shells* (McGraw-Hill., 1959).

[2] S. Timoshenko and J. N. Goodier, *Theory of elasticity* (McGraw-Hill., 1951).

[3] J. M. Monteiro, P. B. Fernandes, F. Vaz, A. R. Pereira, C. A. Tavares, M. T. Ferreira, P. M. Pereira, H. Veiga, E. Kuru, M. S. VanNieuwenhze, Y. V. Brun, S. R. Filipe, and M. G. Pinho, Nature Communications **6** (2015).

[4] J. F. Harvey, *Theory and Design of Pressure Vessels* (Van Nostrand Reinhold, 1985).

[5] X. Zhou, D. K. Halladin, E. R. Rojas, E. F. Koslover, T. K. Lee, K. C. Huang, and J. A. Theriot, Science **348**, 574 (2015).

[6] A. M. Whatmore and R. H. Reed, Journal of General Microbiology **136**, 2521 (1990).

[7] H. H. Tuson, G. K. Auer, L. D. Renner, M. Hasebe, C. Tropini, M. Salick, W. C. Crone, A. Gopinathan, K. C. Huang, and D. B. Weibel, Molecular Microbiology **84**, 874 (2012).

[8] J. Lee, K. Jha, C. E. Harper, W. Zhang, M. Ramsukh, N. Bouklas, T. Dörr, P. Chen, and C. J. Hernandez, ACS Biomaterials Science & Engineering **10**, 2956 (2024).

[9] J. J. Thwaites and N. H. Mendelson, International Journal of Biological Macromolecules **11**, 201 (1989).

[10] E. P. Popov, *Engineering mechanics of solids* (Prentice-Hall International Series in Civil Engineering and Engineering Mechanics, 1990).

[11] V. A. Lund, K. Wacnik, R. D. Turner, B. E. Cotterell, C. G. Walther, S. J. Fenn, F. Grein, A. J. Wollman, M. C. Leake, N. Olivier, A. Cadby, S. Mesnage, S. Jones, and S. J. Foster, eLife **7**, e32057 (2018).

[12] R. G. Bailey, R. D. Turner, N. Mullin, N. Clarke, S. J. Foster, and J. K. Hobbs, Biophysical Journal **107**, 2538 (2014).

[13] L. Pasquina-Lemonche, J. Burns, R. D. Turner, S. Kumar, N. M. Tank, J. S. Wilson, B. Chakrabarti, P. A. Bullough, S. J. Foster, and J. K. Hobbs, Nature **582**, 294 (2020).

[14] A. F. Adedeji-Olulana, K. Wacnik, L. Lafage, L. Pasquina-Lemonche, M. Tinajero-Trejo, J. A. F. Sutton, B. Bilyk, S. E. Irving, C. J. Portman Ross, O. J. Meacock, S. A. Randerson, E. Beattie, D. S. Owen, J. Florence, W. M. Durham, D. P. Hornby, R. M. Corrigan, J. Green, J. K. Hobbs, and S. J. Foster, Science **386**, 573 (2024).
